# Construction and validation of a robust prognostic model based on immune features in sepsis

**DOI:** 10.3389/fimmu.2022.994295

**Published:** 2022-12-02

**Authors:** Yongxin Zheng, Baiyun Liu, Xiumei Deng, Yubiao Chen, Yongbo Huang, Yu Zhang, Yonghao Xu, Ling Sang, Xiaoqing Liu, Yimin Li

**Affiliations:** ^1^ State Key Laboratory of Respiratory Diseases, National Clinical Research Center for Respiratory Disease, Guangzhou Institute of Respiratory Health, Department of Critical Care Medicine, The First Affiliated Hospital of Guangzhou Medical University, Guangzhou, China; ^2^ The First Affiliated Hospital, Guangzhou Medical University, Guangzhou, China

**Keywords:** sepsis, immune, prognostic model, 28-day mortality, immunosuppression

## Abstract

**Purpose:**

Sepsis, with life-threatening organ failure, is caused by the uncontrolled host response to infection. Immune response plays an important role in the pathophysiology of sepsis. Immune-related genes (IRGs) are promising novel biomarkers that have been used to construct the diagnostic and prognostic model. However, an IRG prognostic model used to predict the 28-day mortality in sepsis was still limited. Therefore, the study aimed to develop a prognostic model based on IRGs to identify patients with high risk and predict the 28-day mortality in sepsis. Then, we further explore the circulating immune cell and immunosuppression state in sepsis.

**Materials and methods:**

The differentially expressed genes (DEGs), differentially expressed immune-related genes (DEIRGs), and differentially expressed transcription factors (DETFs) were obtained from the GEO, ImmPort, and Cistrome databases. Then, the TFs-DEIRGs regulatory network and prognostic prediction model were constructed by Cox regression analysis and Pearson correlation analysis. The external datasets also validated the reliability of the prognostic model. Based on the prognostic DEIRGs, we developed a nomogram and conducted an independent prognosis analysis to explore the relationship between DEIRGs in the prognostic model and clinical features in sepsis. Besides, we further evaluate the circulating immune cells state in sepsis.

**Results:**

A total of seven datasets were included in our study. Among them, GSE65682 was identified as a discovery cohort. The results of GSEA showed that there is a significant correlation between sepsis and immune response. Then, based on a P value <0.01, 69 prognostic DEIRGs were obtained and the potential molecular mechanisms of DEIRGs were also clarified. According to multivariate Cox regression analysis, 22 DEIRGs were further identified to construct the prognostic model and identify patients with high risk. The Kaplan–Meier survival analysis showed that high-risk groups have higher 28-day mortality than low-risk groups (P=1.105e-13). The AUC value was 0.879 which symbolized that the prognostic model had a better accuracy to predict the 28-day mortality. The external datasets also prove that the prognostic model had an excellent prediction value. Furthermore, the results of correlation analysis showed that patients with Mars1 might have higher risk scores than Mars2-4 (P=0.002). According to the previous study, Mars1 endotype was characterized by immunoparalysis. Thus, the sepsis patients in high-risk groups might exist the immunosuppression. Between the high-risk and low-risk groups, circulating immune cells types were significantly different, and risk score was significantly negatively correlated with naive CD4+ T cells (P=0.019), activated NK cells (P=0.0045), monocytes (P=0.0134), and M1 macrophages (P=0.0002).

**Conclusions:**

Our study provides a robust prognostic model based on 22 DEIRGs which can predict 28-day mortality and immunosuppression status in sepsis. The higher risk score was positively associated with 28-day mortality and the development of immunosuppression. IRGs are a promising biomarker that might facilitate personalized treatments for sepsis.

## Introduction

Sepsis is a complex disorder that develops as a severe systemic inflammatory response to infection, and is associated with high mortality (1). According to the US report, there were 48.9 (38.9-62.9) million incident cases of sepsis in the world annually and 11.0 (10·1-12·0) million patients died with sepsis (2). Sepsis was recognized as the most expensive burden and threat to human health. Increased mortality was associated with delay in initiating early treatments. The previous study estimated that the survival rate decreases by roughly 10% every hour that appropriate antimicrobial medication is delayed, emphasizing the urgent need for early identification and precise treatments to improve clinical outcomes (1, 3, 4). In 2017, World Health Organization (WHO) also declared that the improvement of sepsis early prevention, early recognition, and treatment is a global health priority (5). Therefore, the identification of septic patients at high risk may help clinicians to screen and identify individuals who are most likely to have poor prognosis, or to detect immunosuppressed states which could benefit from targeted immunostimulating therapies, and eventually improve patients’ prognosis.

Sepsis is an uncontrolled inflammatory response to invasive infection which can disturb homeostasis. After infection, the immune response can eliminate the pathogens but sometimes the host will release damage-associated molecular patterns (DAMPs) to damage organs. However, in late sepsis, sepsis patients have immune suppression which is characterized the lymphocyte exhaustion and the reprogramming of antigen−presenting cells (6, 7). In face of the complex pathophysiology of sepsis and its often challenging clinical evaluation, promising diagnostic biomarkers in sepsis are emerging with the application of blood genomics. Scicluna et al. (8) established endotypes for patients with sepsis through genome-wide blood gene expression profiles. The study provided a method to classify sepsis patients into four different endotypes and the detection of sepsis endotypes may assist in the precise treatments. Besides, increasing studies have identified novel immune biomarkers for early diagnosis and guide immunotherapies in oncology research. The immune related-genes (IRGs) model had been successfully applied in oncology to identify patients at high risk and estimate overall survival (9, 10). However, a robust IRGs model to identify high-risk patients and predict prognosis for adult patients with all-cause sepsis is still lacking.

The primary objective of this study was to construct an IRGs model to predict the prognosis of adult patients with all-cause sepsis. To achieve this aim, we obtain the differentially expressed IRGs (DEIRGs) that we can establish the Cox prediction model based on DEIRGs to predict the patients at high risk and the prognosis for patients with sepsis. Then, we construct a regulatory network between differentially expressed transcription factors (DETFs) and DEIRGs to explore the underlying molecular mechanisms. Besides, we further analyze the immune microenvironment in sepsis patients. Finally, we tested the robustness of the predictive model across the other datasets, and we provided a quantitative tool for predicting the individual probability of death.

## Materials and methods

### Datasets selection, data acquisition, and processing

**Figure 1:**
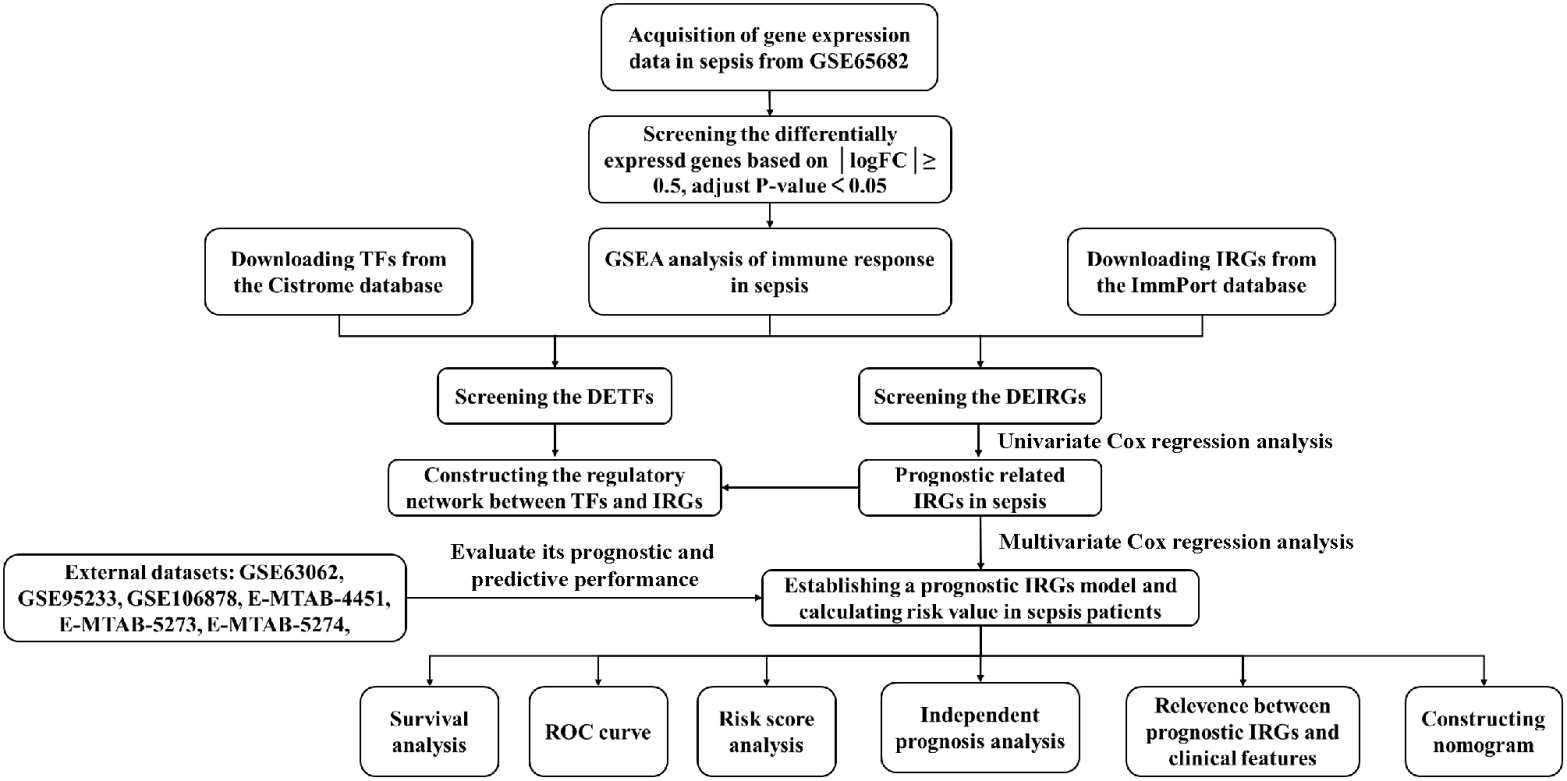
Flowchart of data analysis and validation.

A workflow is shown in **Figure 1**. The Gene Expression Omnibus (GEO) (https://www.ncbi.nlm.nih.gov/geo/) and ArrayExpress (https://www.ebi.ac.uk/arrayexpress/) databases were comprehensively searched from inception to April 2022 to obtain the relevant datasets. The inclusion criteria of datasets were: (1) diagnosis of patients with sepsis; (2) sample size more than 50; (3) age ≥18 years; (4) the endpoints included 28-day mortality; (5) the patient’s specimens were collected before 24h on ICU admission and anti-inflammation treatments. Therefore, the 7 datasets were included in the study. Among them, GSE65682 was recognized as a training set because it was a large cohort study, and the other datasets were retained for model validation. The details of these datasets are shown in **Table 1**. To identify IRGs, we downloaded 2,483 IRGs from the Immunology Database and Analysis Portal (ImmPort) (http://www.immport.org/). Moreover, to construct the regulatory network, we obtained the transcription factors (TFs) from Cistrome Project (http://www.cistrome.org/).

**Table 1:** Basic information of the datasets included in this study.

Accession	Study population	Sample type	Country	Timing of gene expression profiling	Mortality / Total patients
GSE65682	Patient diagnoses sepsis due to cap, hap and non-infectious control.	Blood	Netherlands and England	On ICU admission	114 / 802
GSE63042	Patients with SIRS or sepsis	Blood	America	The day of enrollment upon presentation to the ED.	28 / 129
GSE95233	Patients with septic shock and healthy volunteers	Blood	France	Day 1 of ICU admission	34 / 124
GSE106878	septic shock patients from the CORTICUS-trial	Circulating leukocytes	International	Before hydrocortisone application	26 / 94
E-MTAB-4451	Patients with severe sepsis due to CAP	Circulating leukocytes	England	On ICU admission	52 / 106
E-MTAB-5273	Patients with sepsis due to CAP or faecal peritonitis.	Circulating leukocytes	England	First day of ICU stay	43 / 221
E-MTAB-5274	Patients with sepsis due to CAP or faecal peritonitis.	Circulating leukocytes	England	First day of ICU stay	14 / 106

### Differential expression analysis in sepsis

All the genes in GSE65682 were differentially analyzed by using limma R packages (http://www.bioconductor.org/packages/release/bioc/html/limma.html) (11). The parameter for DEGs screened was│Log2Foldchange│≥0.5 and P-value < 0.05. The Volcano plots were drawn by ‘ggplot2’ R package. Then, the IRGs that were overlapping with DEGs were identified as DEIRGs. Similarly, DETFs were obtained by matching TFs with DEGs.

### DEGs and gene set enrichment analysis

GSEA was used to assess related pathways and molecular mechanisms in sepsis. We performed the GSEA by the R package ‘clusterProfiler’. Normalized enrichment score (NES) and false discovery rate (FDR) were used to quantify enrichment magnitude and statistical significance, respectively (12, 13).

### Identification of prognostic DEIRGs and construction of the regulatory network

To identify the prognostic DEIRGs (P <0.01), R package ‘survival R’ was used to perform univariate Cox regression analysis. Then, it is important to further explore the mechanisms of TFs to regulate the prognostic DEIRGs. Thus, we further analyzed the coexpression relationship between TFs and prognostic DEIRGs by calculating Pearson’s correlation coefficient. A regulatory network was constructed based on the filter thresholds (P value <0.001 and |cor| > 0.5). The network was visualized by using Cytoscape software.

### Construction of the prognostic prediction model in sepsis and development of nomogram

Based on the univariate Cox regression analysis, prognostic DEIRGs were recognized as the biomarkers for multivariate Cox regression analysis. According to the median risk score value, conducted between low-risk and high-risk groups by using ‘survival’ R package. To evaluate the sensitivity and specificity of the prediction model, the receiver operating characteristics (ROC) curve was calculated using the ‘survivalROC’ package. The area under the ROC curve (AUC) was used to evaluate the prognostic model: 0.5-0.7 (moderate), 0.7-0.8 (better), and >0.9 (excellent).

To provide a quantitative tool for predicting probability of 28-day mortality in septic patients, we construct a nomogram according to the DEIRGs in the prognostic model and clinic features. The patients' clinic features are shown in **Supplementary Table 1**.

### Validation in multiple external datasets

To evaluate the predictive performance of the prognostic model, 6 datasets (GSE63062, GSE95233, GSE106878, E-MTAB-4451, E-MTAB-5273, and E-MTAB-5274) were included according to the inclusion criteria. The prognostic model was used to predict the 28-day mortality of external datasets. Furthermore, the ROC curve was generated to determine sensitivity and specificity in the prognostic model.

### Gene ontology and pathway enrichment analysis for DEIRGs in the prognostic model

To explore the mechanisms and functions of DEIRGs in the prognostic model, we performed Gene Ontology (GO) and Pathway Enrichment Analysis (KEGG) through the DAVID database (https://david.ncifcrf.gov/). Upon GO analysis and KEGG analysis, a P value <0.05 was recognized as statistical significance. The results of GO analysis were classified into three functional groups: biological process (BP), molecular function (MF), and cellular component (CC).

### Correlation analysis between clinical features and DEIRGs in prognostic model

The correlation between risk score, gene expression value, and clinical features (age, gender, diabetes, ICU acquired infection (ICUA), and endotype class) were analyzed by using the ‘beeswarm’ R package. A P value <0.05 indicated statistical significance.

### Exploration of circulating immune cells between low-risk and high-risk groups

CIBERSORTx (https://cibersort.stanford.edu/), an online analytical tool based on a kind of deconvolution algorithm iterated 1000 times, was available to provide an estimation of the abundances of member cell types in a mixed cell population by using gene expression data (14). Then, the content of 22 types of circulating immune cells in each sample was visualized by a vertical stack bar. Furthermore, the difference analysis of immune cells between low-risk and high-risk groups was shown by drawing barplot diagrams. Additionally, we explored the correlation between immune cells and risk score by Spearman correlation analyses. A P value <0.05 was considered statistical significance.

### Statistical analysis

All statistical analyses were performed using R software and Grapad prism 9.0. The ‘limma R’ package was used to conduct differential expression analysis. The R package ‘clusterProfiler’ was adopted for assessing related pathways and molecular mechanisms in sepsis. The prognostic prediction model was constructed by univariate and multivariate Cox regression analysis. Besides, the ‘survival’, ‘survival ROC’, and ‘risk Plot’ R packages were applied to evaluate the survival difference between the high-risk and low-risk groups and assess the sensitivity and specificity in the prognostic model. Then, the ‘beeswarm’ R package was used to explore the correlation between clinical features and DEIRGs in the prognostic model. P value < 0.05 was considered statistically significant.

## Results

### DEGs, DEIRGs, DETFs and GSEA analysis

**Figure 2:**
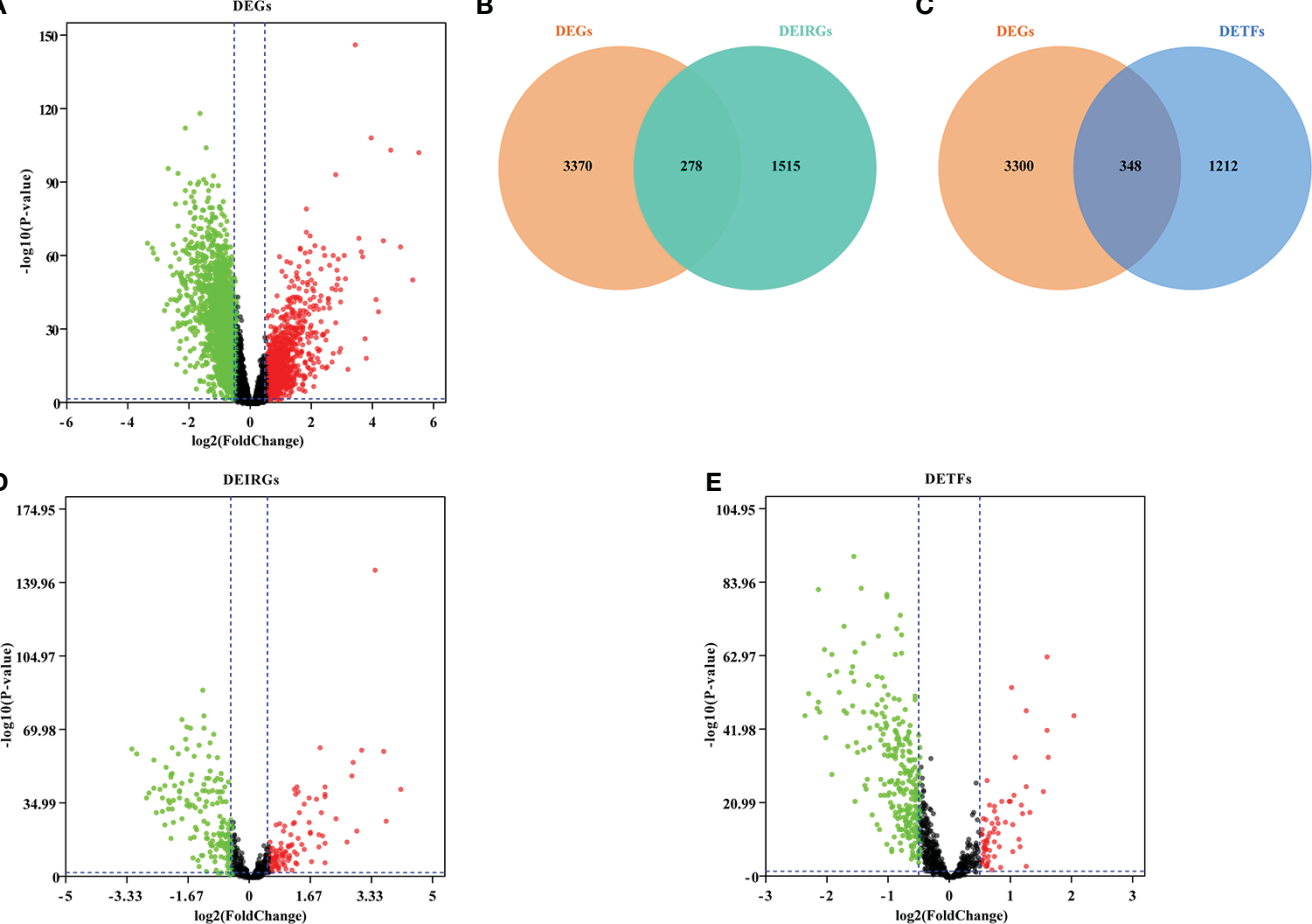
Screening DEGs, DEIRGs and DETFs. **(A)** Volcano plot showing DEGs in GSE66890; **(B)** Venn diagram showed DEIRGs; **(C)** Venn diagram showed DETFs; **(D)** Volcano plot showing DEIRGs; **(E)** Volcano plot showing DETFs. Based on the |fold change|>0.5 and FDR<0.05, the red points represent upregulated genes and the green points represent downregulated genes. No significant differences are showed in black.

After the differential expression analysis of GSE65682, we obtained 3,648 DEGs (FDR <0.05, │Log2FC│≥0.5) (**Figure 2A**; **Supplementary Table 2**). To identify the DEIRGs, we downloaded all 2,483 immune genes from the ImmPORT database. Then, we matched IRGs with DEGs and obtained 278 DEIRGs (**Figures 2B, D**; **Supplementary Table 3**). Similarly, we searched and downloaded all 1,560 TFs from the Cistrome database. We matched TFs with DEGs and obtained 348 DETFs (**Figures 2C, E**; **Supplementary Table 4**).

**Figure 3:**
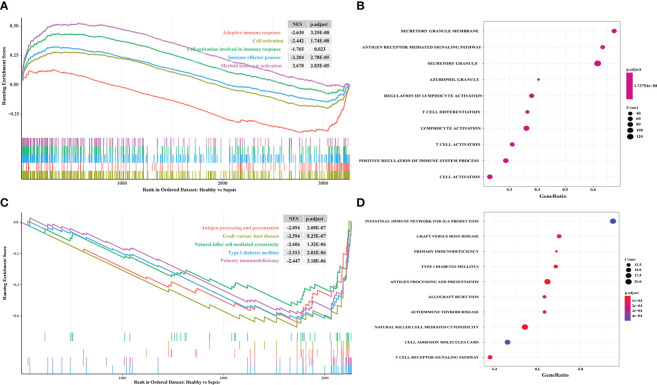
Exploring the difference of immune response between sepsis and healthy by using GSEA. **(A)** The enriched gene sets in GO collection; **(B)** The results of GO analysis from GSEA; **(C)** The enriched gene sets in KEGG collection; **(D)** The results of KEGG analysis from GSEA. The enrichment score of curve above 0 points indicates that the gene sets were activated in healthy. The curve below 0 points indicates that the gene sets were activated in sepsis. p.adjust, adjusted p-value; NES, normalized enrichment score.

In order to explore the immune response in sepsis, we downloaded the KEGG gene sets and all GO gene sets from MsigDB. Then, the changes of these pathways and functions in gene sets were analyzed, namely Healthy vs Sepsis. According to our analysis, we found that immune response played an important role in the development of sepsis. In GO gene sets, we found that adaptive immune response was significantly upregulated in sepsis. However, cell activation involved in immune response, immune effector process, and myeloid leukocyte activation were upregulated in healthy and sepsis (**Figures 3A, B**). In KEGG gene sets, antigen processing and presentation, natural killer cell-mediated cytotoxicity, and primary immunodeficiency were most significantly increased (**Figures 3C, D**). Our results showed that there is a significant correlation between sepsis and immune response, and provide a theoretical basis for the construction of immune genes model to predict the prognosis of sepsis patients.

### Identification of prognostic DEIRGs and construction of regulatory network

**Figure 4:**
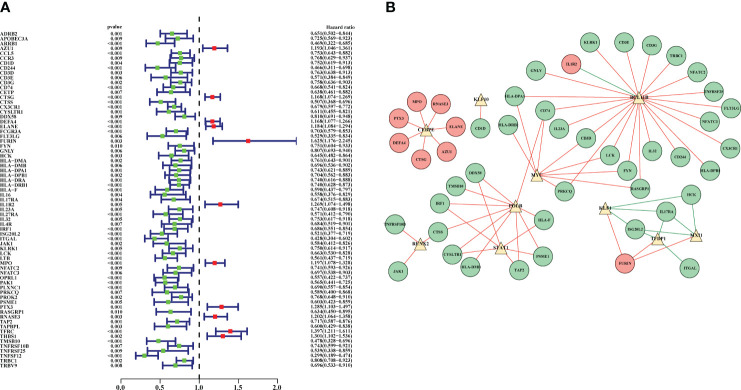
Prognostic DEIRGs and regulatory network between DETFs and prognostic DEIRGs. **(A)** Forest plot for prognostic DEIRGs in sepsis. Red and green dots were recognized as high-risk and low-risk, respectively; **(B)** Regulatory network between prognostic DEIRGs and DETFs. The red and green circles indicate high-risk DEIRGs and low-risk DEIRGs, respectively. The yellow triangles were applied to symbolize the DETFs. Moreover, the red and green lines were used to indicate a positive and negative correlation between prognostic DEIRGs and DETFs.

Univariate Cox regression analysis was applied to screen and identify the prognostic genes in sepsis. According to P value <0.01, 69 prognostic DEIRGs were obtained (**Figure 4A**; **Supplementary Table 5**). Among them, 11 prognostic DEIRGs were high-risk and the others were low-risk. Then, to explore the molecular mechanisms between DETFs and prognostic DEIRGs, a regulatory network between DETFs and prognostic DEIRGs was constructed (**Figure 4B**; **Supplementary Table 6**). A total of 69 prognostic DEIRGs and 10 TFs were shown in the regulatory network (**Figure 4B**). As shown in the regulatory network, almost all expression level of high-risk DEIRGs (MPO, PTX3, DEFA4, CTSG, AZU1, ELANE, and RNASE3) were upregulated by CEBPE. Additionally, IL1R2 was upregulated by BCL11B, and FURIN was regulated by KLF1, TFDP1, and MX11. Besides, most low-risk DEIRGs had a positive relationship with BCL11B, MYC, POLB, STAT1, RUNX2, and KLF10. The other low-risk DEIRGs (HCK, IL17RA, ISG20L2, and ITGAL) were negatively regulated by KLF1, TFDP1, and MX11. The coefficient filter >0.5 and the P value <0.001 were set as the threshold to indicate statistical significance.

### Construction of prognostic prediction model in sepsis

The 69 prognostic DEIRGs were obtained by univariate Cox regression analysis. Then, these prognostic DEIRGs were further incorporated into multivariate Cox regression analysis. Finally, 22 DEIRGs might serve to be the prognostic factors to independently predict the prognosis of sepsis patients (**Table 2**). Thus, the expression profiles of 22 DEIRGs were applied to construct the prognostic model to predict the 28-day mortality in sepsis patients. To obtain the survival risk score, the expression value and relative coefficients of 22 DEIRGs were used to calculate. The formulas was shown in **Supplementary Table 7**. Based on the median risk score value, 479 septic patients were classified into a high-risk group (n= 239) and a low-risk group (n=240) (**Supplementary Table 7**).

**Table 2:** Multivariate Cox regression analyses of 22 IRGs of risk model in sepsis.

DEIRGs	coef	HR	HR.95L	HR.95H	pvalue
ADRB2	-0.693479	0.499834	0.3647514	0.6849434	1.60E-05
CD1D	-0.394755	0.6738449	0.4833991	0.9393211	0.0198413
CD74	0.7029737	2.0197499	1.102899	3.6987881	0.0227718
CETP	-1.176492	0.3083585	0.2045131	0.4649334	1.96E-08
ELANE	0.3804079	1.4628811	0.9891965	2.1633934	0.0567083
FYN	0.4151731	1.5146329	0.9758725	2.3508326	0.0641593
GNLY	-0.244232	0.7833063	0.6309512	0.9724504	0.0268901
HLA-DRA	-0.761003	0.4671977	0.2847489	0.7665478	0.0025925
IL16	1.0580916	2.8808678	1.2936444	6.4155183	0.0095908
IL17RA	-0.54816	0.5780126	0.3256761	1.0258613	0.0611051
IL1R2	0.2860863	1.3312073	1.0111484	1.7525746	0.0414519
LTB	-0.651796	0.5211092	0.3413782	0.7954661	0.0025252
MPO	-0.615383	0.540434	0.3689694	0.7915804	0.0015765
PLXNC1	-0.488971	0.613257	0.3999037	0.9404368	0.0249953
PSME1	0.7359292	2.0874208	0.9682696	4.500116	0.0604232
TAP2	-0.386602	0.6793615	0.453822	1.0169892	0.0603643
TFRC	0.1729455	1.1888014	0.9450299	1.4954539	0.1396536
THBS1	0.3465734	1.4142134	1.1220993	1.782373	0.0033265
TNFRSF10B	0.7411038	2.0982502	1.4481151	3.0402651	8.97E-05
TNFSF12	-0.898271	0.4072731	0.230409	0.7198997	0.0019965
TRBV9	-0.336649	0.7141593	0.4942263	1.0319634	0.0730621
DEFA4	0.1906303	1.210012	0.9676379	1.513096	0.0946212

**Figure 5:**
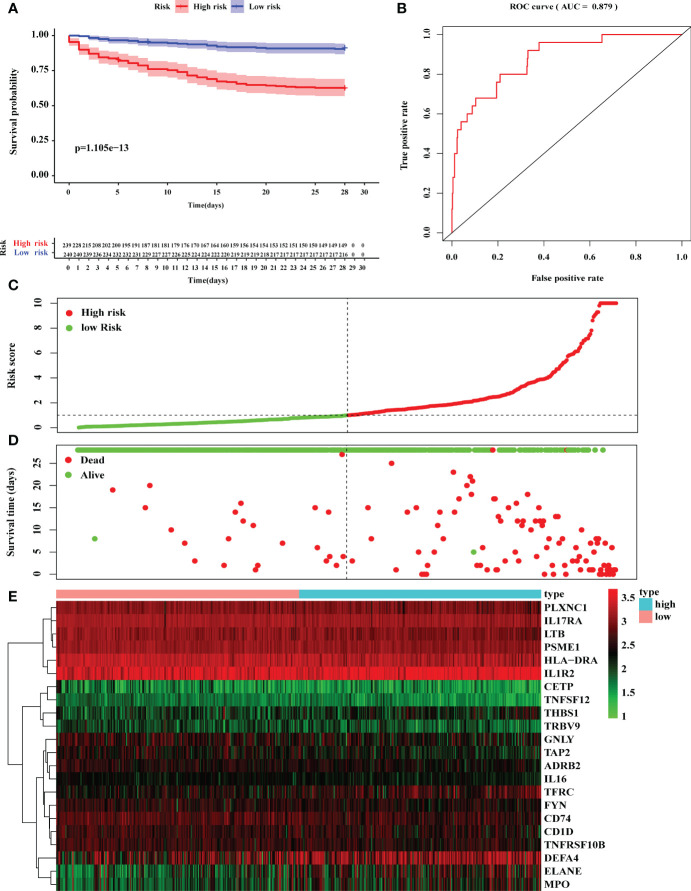
Construction of prognostic model based on 22 DEGs. **(A)** Kaplan–Meier survival analysis of 28-day mortality between high-risk groups (red) and low-risk groups (blue). The color of each survival line indicated the 95% CI of probability of survival at each time point. **(B)** The ROC curve showed the AUC value of prognostic model. **(C)** The risk score analysis between high-risk and low-risk groups. **(D)** The survival status analysis between high-risk and low-risk groups. **(E)** The differentially expression analysis of 22 DEIRGs in prognostic model from 479 sepsis patients.

Then, Kaplan–Meier survival analysis was performed to analyze the 28-day mortality of high-risk groups (n= 239) and low-risk groups (n=240). As expected, the 28-day mortality of high-risk groups was significantly higher than low-risk group (**Figure 5A**; **Supplementary Table 8**). Furthermore, we drew an ROC curve to evaluate the sensitivity and specificity of the prognostic model. The results showed that the AUC value was 0.879 which symbolized that the prognostic model had a better accuracy to predict the 28-day mortality of high-risk and low-risk groups (**Figure 5B**). Additionally, the riskscope curve was constructed (**Figure 5C**) and the survival status of the two groups is shown in **Figure 5D**. The differential expression analysis of 22 DEIRGs are shown in **Figure 5E**.

### Validation of prognostic model by external datasets

**Figure 6:**
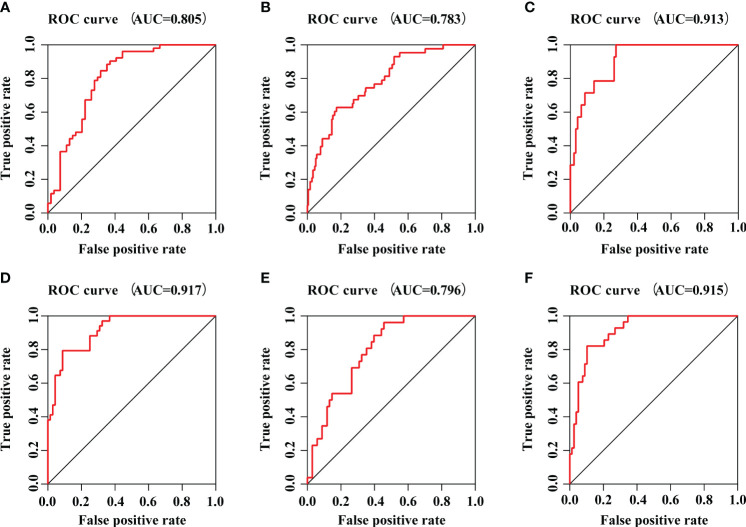
The prognostic efficacy of IRGs prognostic model. **(A)** The ROC curve of E-MTAB-4451 dataset. **(B)** The ROC curve of E-MTAB-5273 dataset. **(C)** The ROC curve of E-MTAB-5274 dataset. **(D)** The ROC curve of GSE95233 dataset. **(E)** The ROC curve of GSE106878 dataset. **(F)** The ROC curve of GSE63042 dataset.

To further evaluate the accuracy and reliability of the prognostic model, six datasets in line with the inclusion criteria were chosen to perform external validation. ROC analysis was performed to investigate the prognostic value of the prediction model. The AUC was 0.805 in E-MTAB-4451 (**Figure 6A**), AUC was 0.783 in E-MTAB-5273 (**Figure 6B**), AUC was 0.913 in E-MTAB-5274 (**Figure 6C**), AUC was 0.917 in GSE95233 (**Figure 6D**), AUC was 0.796 in GSE106878 (**Figure 6E**) and AUC was 0.915 in GSE63042 (**Figure 6F**), respectively. Therefore, the IRGs prognostic model had an excellent prediction value.

### Independent prognosis analysis and exploring the relationships between DEIRGs in prognostic model and clinical features in sepsis

**Figure 7:**
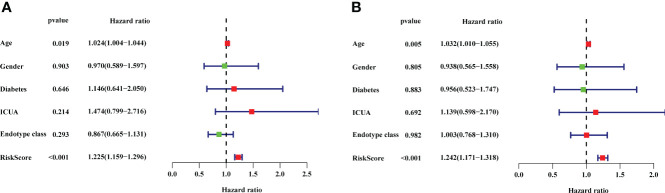
The results of univariate independent prognostic analysis and multivariate independent prognostic analysis. **(A)** Univariate independent prognostic analysis. **(B)** Multivariate independent prognostic analysis. The red dots and green dots in the forest map indicated that the clinical feature was a high-risk factor and low-risk factor, respectively. ICUA, ICU acquired infection.

The 22 DEIRGs in the prognostic model had a better predictive ability to investigate the 28-day mortality in sepsis. Then, we further conducted the univariate independent prognostic analysis and multivariate independent prognostic analysis to explore the correlation between clinical features and 28-day mortality in sepsis. The results of the univariate independent prognostic analysis showed that age (P = 0.019) and risk score (P <0.001) were related to the 28-day mortality, respectively (**Supplementary Table 9**; **Figure 7A**). The results of the multivariate independent prognostic analysis also showed that age (P = 0.005) and risk score (P <0.001) were the independent prognostic factors to predict the 28-day mortality in sepsis (**Supplementary Table 10**; **Figure 7B**).

**Figure 8:**
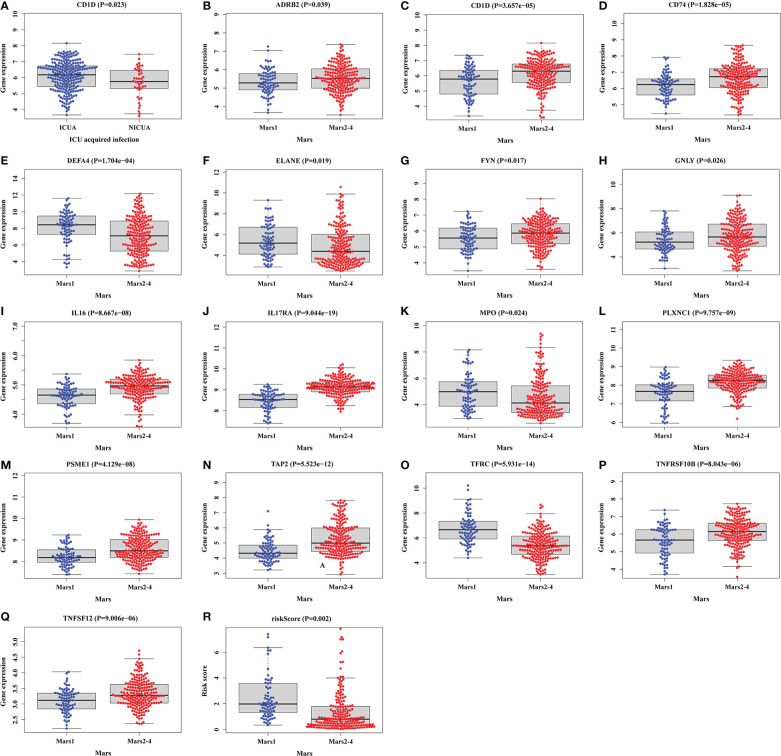
Relationships between clinical features and DEIRGs in prognostic model. **(A)** Different expression of CD1D between the ICUA/NICUA in sepsis. **(B–R)** Different expression of DEIRGs in prognostic model between Mars1 and Mars2-4 in sepsis. ICUA, ICU acquired infection; NICUA, No ICU acquired infection; Mars, molecular diagnosis and risk stratification of sepsis.

Then, the correlation between clinical features and DEIRGs in the prognostic model was further explored (**Supplementary Material 11**). Among the clinical features, the endotype class was classified into four classes, including Mars1, Mars2, Mars3, and Mars4 (8). According to the research, the Mars1 endotype was characterized by a pronounced decrease in the expression of genes corresponding to key innate and adaptive immune cell functions such as Toll-like receptor, nuclear factor κB (NFκB1) signaling, antigen presentation, and T-cell receptor signaling, which might be characterized by immune paralysis. The other endotypes (Mars2-4) were characterized by high expression of genes involved in pro-inflammatory (eg, NF-κB signaling) and innate (eg, interferon signaling) immune reactions, which are characterized as pro-inflammatory and innate immune response. As shown in **Figure 8**, the expression level of CD1D was higher in ICU-acquired infection (ICUA). Besides, the expression levels of ADRB2, CD1D, CD74, FYN, GNLY, IL16, IL17RA, PLXNC1, PSME1, TAP2, TNFRSF10B and TNFSF12 in Mars2-4 were significantly higher than Mars1. The expression levels of DEFA4, ELANE, MPO, and TFRC were significantly lower in Mars1 compared to those in Mars2-4. Therefore, DEFA4, ELANE, MPO, and TFRC might be related to immune paralysis in sepsis. Additionally, patients with Mars1 might have higher risk scores than Mars2-4 (**Figure 8R**) which was consistent with the results of Scicluna et al. (8).

### Development of nomogram to predict the 28-day mortality in sepsis

**Figure 9:**
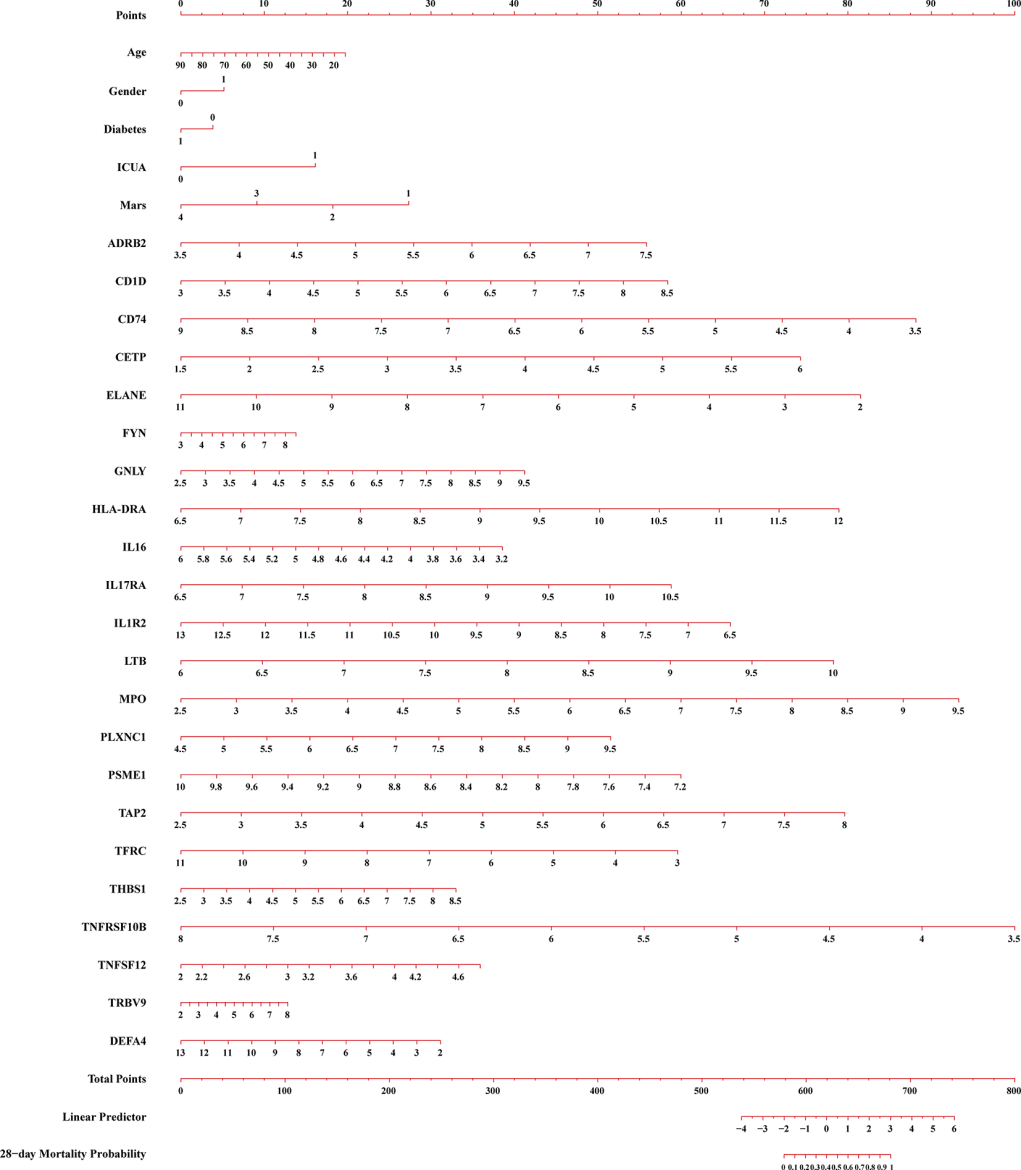
A constructed nomogram for 28-day mortality prediction of a patients with sepsis. ICUA, ICU acquired infection; Mars, molecular diagnosis and risk stratification of sepsis.

We constructed a nomogram to predict the 28-day mortality in sepsis according to clinical features and DEIRGs in the prognostic model (**Figure 9**). The value of each of the variables was given a score based on the points scale axis. The total score was calculated by adding each single score. Then, the total points were projected to the 28-day mortality probability scale axis to estimate the probability of death in sepsis.

### Functional analysis for DEIRGs in prognostic model

**Figure 10:**
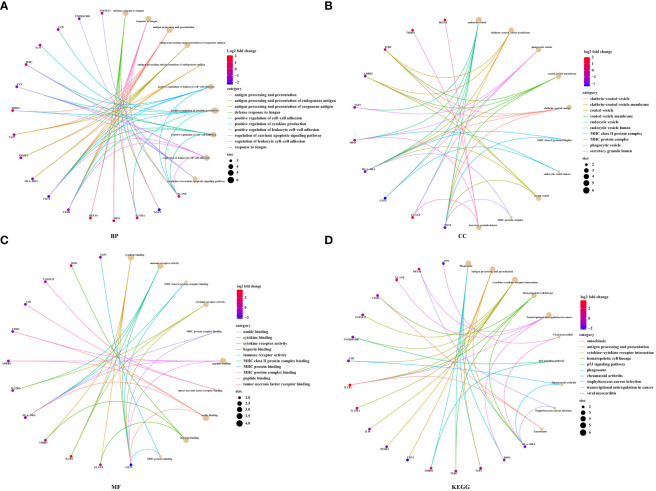
The functional enrichment analysis for DEIRGs in prognostic model. **(A)** Biological process. **(B)** Cell component. **(C)** Molecular function. **(D)** KEGG pathway enrichment analysis.

To explore the functional changes for DEIRGs in the prognostic model, we performed the functional enrichment analysis. The GO terms were divided into three functional groups, including biological process (BP), cell component (CC), and molecular function (MF). The top 10 significant enrichment results are shown in **Figure 10**. In BP groups, DEIRGs were mainly enriched in antigen processing and presentation, positive regulation of cytokine production and positive regulation of leukocyte cell−cell adhesion (**Figure 10A**). In CC groups, DEIRGs were mainly involved in MHC class II protein complex, MHC protein complex and phagocytic vesicle (**Figure 10B**). In MF groups, DEIRGs mainly enriched in cytokine binding, cytokine receptor activity and immune receptor activity (**Figure 10C**). As for the KEGG analysis, DEIRGs were mainly involved in antigen processing and presentation, cytokine−cytokine receptor interaction and hematopoietic cell lineage (**Figure 10D**).

### Correlation analysis between DEIRGs and circulating immune cells

**Figure 11:**
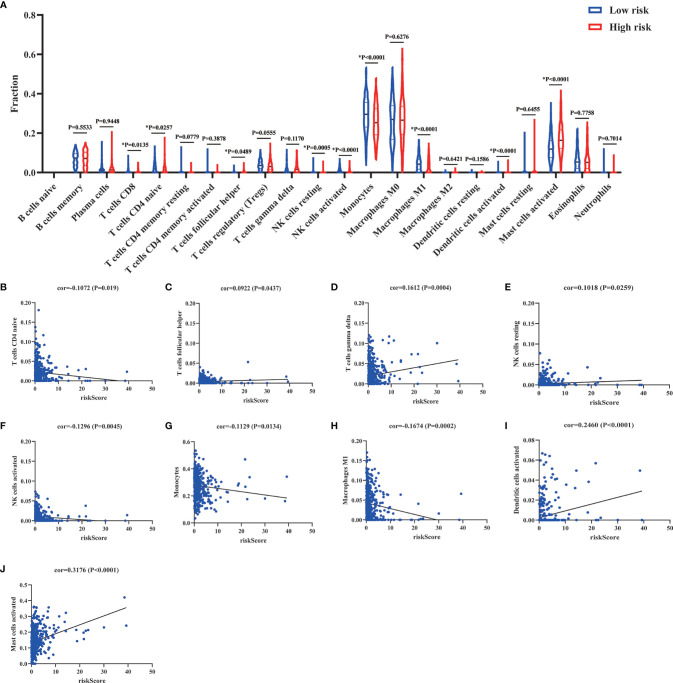
Comparison and correlation of circulating immune cells between low-risk and high-risk groups. **(A)** Comparison of circulating immune cells between low-risk and high-risk groups *via* CIBERSORTx. **(B–J)** Correlation between risk scores and circulating immune cells *via* Spearman correlation analysis. *p < 0.05.

Numerous studies had demonstrated that circulating immune cells levels were associated with the prognosis of patients (15, 16). Therefore, we wanted to explore the different status of circulating immune cells between low-risk and high-risk groups. As shown in **Supplementary Figure 1**, the status of immune cells was significantly different in low-risk groups compared to the high-risk groups. Then, we further analyzed the composition of immune cells between low-risk and high-risk groups. The results of CIBERSORTx demonstrated that compared to the high-risk groups, CD8+ T cells (P=0.0135), resting (P=0.0005), and activated NK cells (P<0.0001), monocytes (P<0.0001), and M1 macrophages (P<0.0001) were more abundant in low-risk groups, while naive CD4+ T cells (P=0.0257), follicular helper T cells (P=0.0489) and activated dendritic cells (P<0.0001) were significantly enriched in high-risk groups (**Figure 11A**). Besides, we also analyzed the correlation of risk score and 22 immune cell types *via* Spearman correlation analyses. The results showed that risk scores were significantly positively correlated with follicular helper T cells (P=0.0437), gamma delta T cells (P=0.0004), resting NK cells (P=0.0259), activated dendritic cells (P<0.0001), and activated mast cells (P<0.0001), whereas were significantly negatively correlated with naive CD4+ T cells (P=0.019), activated NK cells (P=0.0045), monocytes (P=0.0134) and M1 macrophages (P=0.0002).

## Discussion

Sepsis, with high heterogeneity, is characterized by aberrant immune responses, including hyperinflammation and immune suppression (17). Increasingly, studies have pointed out that IRGs are promising novel biomarkers that may have important predictive and prognostic value (8, 18, 19). Thus, our research demonstrates that immune response played an important role in the development of sepsis. Then, the Cox prediction model obtained the 22 DEIRGs to classify the patients into low-risk and high-risk groups and construct the prognostic model. The regulatory network between TFs and prognostic DEIRGs was constructed to reveal the potential novel molecular mechanisms in sepsis. In this study, the prognostic model had a better accuracy to predict the 28-day mortality in sepsis. The external datasets also validated that the prognostic model had an excellent prediction value. Besides, we further developed a nomogram to provide a tool for predicting the probability of 28-day mortality in sepsis. We further explore the functional changes *via* functional enrichment analysis. Finally, the circulating immune cells were evaluated by CIBERSORTx.

As we know, the biomarkers to diagnose and predict the prognosis of sepsis were lacking due to the complex pathogenesis and high heterogeneity in sepsis. The unbalanced immune response of sepsis was initially activated to release tremendous damage-associated molecular patterns (DAMPs) such as cytokines. The cytokine storm will further lead to organ damage and even death (20). However, longitudinal analyses of immune response showed that patients developed persistent inflammation and immunosuppression in the late stage of sepsis (21). Therefore, the aberrant immune responses during sepsis might reflect the disease progression. The results of GSEA in this study also showed that immune responses were significantly related to the development of sepsis (**Figure 3**).

IRGs, promising novel biomarkers, had been used to predict the prognosis in many diseases (22). Even in sepsis, Lu et al. demonstrated the immune genes exhibited superior diagnostic and predictive efficacy in mortality than clinical characteristics (18). However, the molecular mechanisms of prognostic DEIRGs in sepsis were still unclear. In our study, we have identified 11 prognostic DEIRGs with high risk and 58 prognostic DEIRGs with low risk *via* univariate Cox regression analysis (**Figure 4A**). TFs, as an enhancer or promoter, could regulate the genes’ expression by binding to a particular DNA region. The regulatory network was constructed between TFs and prognostic DEIRGs (**Figure 4B**). We found key TFs (CEBPE, BCL11B, MYC, POLB, and STAT1) which had the most downstream DEIRGs and relatively high correlation coefficients. CEBPE has been demonstrated to be involved in the generation and proliferation of neutrophils (23). Besides, CEBPE was an important target to promote the innate immune system (e.g. neutrophil) to kill the bacteria (24). In this study, almost high-risk DEIRGs (MPO, PTX3, DEFA4, CTSG, AZU1, ELANE, and RNASE3) were upregulated by CEBPE which indicated that these high-risk DEIRGs might promote the inflammation and innate immune responses. Additionally, the BCL11B gene was essential for T cell and NK cell development and function (25). MYC, POLB, and STAT1 have also been described as having a strong relationship to the functions of the immune system and the clearance of pathogens (26–28). Therefore, most low-risk DEIRGs had a positive relationship with BCL11B, MYC, POLB and STAT1 might have an important role in regulating immune responses and defending against pathogens.

To construct the prognostic model, we further conducted the multivariate Cox regression analysis to identify the prognostic DEIRGs (**Table 2**). Then, the prognostic model could predict the 28-day mortality in sepsis with better accuracy (**Figures 5B**, **6**). Among them, we obtained 4 high-risk genes (ELANE, IL1R2, MPO, and DEFA4) and 18 low-risk genes. ELANE and MPO had been demonstrated to involve in neutrophil protease activity. The expression levels of ELANE and MPO were correlated directly with organ failure and mortality which was in line with our results (29, 30). Besides, IL1R2, a decoy receptor for IL-1, has been implicated in sepsis (31). Previous studies have proven that IL1R2 was a biomarker to distinguish septic shock from non-septic shock postsurgical patients. The high expression of IL1R2 was significantly correlated to death in patients with postsurgical shock (32, 33). Interestingly, Liang et al. (34) pointed out that IL1R2 could distinguish gram-negative/gram-positive bacterial infection. The elevation of serum IL1R2 could be a biomarker to diagnose septic patients infected by gram-negative bacteria.

As we know, numerous studies have provided prognostic models/biomarkers for predicting overall survival in sepsis. However, these predictive factors were not applied to all sepsis patients due to the high heterogeneity. Thus, it is critical to stratify patients to guide treatments. A VANISH randomized trial categorized patients into SRS (sepsis response signatures) 1 and SRS2 according to transcriptomic profile. Patients with the immunocompetent SRS2 endotype might have significantly higher mortality when treated with corticosteroids than with placebo (35). Additionally, Scicluna et al. (8) classified patients with sepsis into four different endotypes (Mars1, Mars2, Mars3 and Mars4) upon ICU admission. According to the research, Mars1, with TAP2 transcripts denoting, was characterized by immune paralysis and poor prognosis, whereas Mars2-4 were characterized by high expression of pro-inflammatory genes. Our research also demonstrated that TAP2 was significantly downregulated in Mars1 compared to Mars2-4 (**Figure 8N**). TAP2 was a subunit of major histocompatibility complex class I (MHC-I) molecules involved in antigen processing (36). TAP2 has the potential to inhibit lipopolysaccharide-induced proinflammation by negative regulation of toll-like receptor-4 (TLR4) (37). Besides, our research also showed that most sepsis patients with high risk might have the Mars1 endotype which indicated the poor prognosis of patients with the Mars1 endotype (**Figure 8R**). This result was also in line with the previous study. Therefore, patients with sepsis in immunosuppression might be associated with an increased risk of mortality.

As we know, patients who survive early sepsis often develop a hypoinflammatory state and nosocomial infections which lead to high mortality (7, 17). Immune suppression in patients with sepsis is characterized by enhanced apoptosis of immune cells, T cell exhaustion, and reduced expression of activating cell surface molecules. Previous studies have proven that T cell exhaustion in immunosuppression was related to poor outcomes (38). The apoptosis of T cells (CD4+, CD8+, and Th17) will result in immunosuppression and is associated with higher mortality (39). Besides, nature killer (NK) cells could clear the pathogens and promote inflammation through the production of IFN-γ. However, NK cells will become tolerant and cytokine production of IFN-γ and TNF-α will be impaired in the late stage of sepsis. The proportion of NK cells in lymphocytes was negatively associated with 28-day mortality in septic patients (40, 41). Monocytes and macrophages are important components of the immune system that can remove pathogens and contribute to the immune response by antigen presentation. The M1 macrophages were characterized by the production of proinflammatory cytokines and antimicrobial activity. However, the polarization of M1 macrophages will be inhibited in immunosuppression. The M1 macrophage reprogramming will develop a pathological anti-inflammatory response to sepsis and increase the risk of immunosuppression (42, 43). In our research, CD8+ T cells (P=0.0135), resting (P=0.0005) and activated NK cells (P<0.0001), monocytes (P<0.0001), and M1 macrophages (P<0.0001) were more abundant in low-risk groups which indicated a hyperinflammatory state in low-risk groups (**Figure 11A**). Besides, the results of correlation analyses also showed that risk scores were significantly negatively correlated with naive CD4+ T cells (P=0.019), activated NK cells (P=0.0045), monocytes (P=0.0134), and M1 macrophages (P=0.0002) (**Figure 11**). In toto, the patients with high risk scores might be associated with immunosuppression. The risk score was positively associated with the development of immunosuppression in sepsis. The risk score might provide assistance for distinguishing sepsis patients with immunosuppression.

However, in spite of the remarkable results, there are several limitations that we could not ignore. First, a lot of publicly available sepsis datasets were excluded for lacking the mortality outcome. These datasets might concentrate on the differential diagnosis or other poor outcomes. The exclusion of these datasets might cause potential selection bias. Second, our prognostic model had a better performance in distinguishing patients with high risk, evaluating 28-day mortality in sepsis, and identifying sepsis patients with immunosuppression. However, it still needs large prospective cohorts to validate the performance before the prognostic model was applied to general use. Third, the datasets we included did not provide the details of comorbidities or other diseases. Therefore, we can’t exclude the impact of these factors on the prognostic model. Fourth, it may not accurately identify the immune cell types in sepsis according to bulk RNA-Seq data and the CIBERSORTx deconvolution algorithm. It still required further experiments (e.g. Flow Cytometry) to validate the results. Finally, the *vivo* and *vitro* experiments may help us identify the hub genes to predict the prognosis of sepsis and identify the patients with immunosuppression.

## Conclusion

Our study demonstrated that immune response played an important role in the development of sepsis. IRGs, as promising novel biomarkers, were used to construct the TFs-DEIRGs regulatory network and prognostic prediction model, respectively. The TF-DEIRGs regulatory network has revealed the potential molecular mechanisms for DEIRGs in sepsis. The prognostic model, with great performance, could identify the patients with high risk and predict the 28-day mortality in patients with sepsis. Besides, the prognostic DEIRGs were also related to the immune cell circulating and immunosuppression state, which might promote individualized therapy for sepsis patients.

## Data availability statement

The datasets presented in this study can be found in online repositories. The names of the repository/repositories and accession number(s) can be found in the article/**Supplementary Material**.

## Author contributions

YoZ and YH analysed and interpreted the data. YoZ, YuZ, YC and BL performed the bioinformatics analyses. XD, YX and LS performed data acquisition and figure preparations. YoZ and YH wrote the manuscript. XL and YL revised and edited this paper. All authors contributed to the article and approved the submitted version.

## Funding

This work was supported by the National Natural Science Foundation of China (81870069, 81970071, 82070084, 82270085), the Natural Science Foundation of Guangdong Province (2020A1515011459, 2021A1515012565), the Science and Technology Program of Guangzhou (202102010366, 202201020444), the State Key Laboratory of Respiratory Disease Independent Program (SKLRD-Z-202108), Guangdong Marine Economy Development Special Project (GDNRC[2022]35).

## Conflict of interest

The authors declare that the research was conducted in the absence of any commercial or financial relationships that could be construed as a potential conflict of interest.

## Publisher’s note

All claims expressed in this article are solely those of the authors and do not necessarily represent those of their affiliated organizations, or those of the publisher, the editors and the reviewers. Any product that may be evaluated in this article, or claim that may be made by its manufacturer, is not guaranteed or endorsed by the publisher.

## Supplementary material

Supplementary Table 1 The clinic features of GSE65682.

Supplementary Table 2 The differential expression analysis of GSE65682.

Supplementary Table 3 The differential expression analysis of IRGs.

Supplementary Table 4 The differential expression analysis of TFs.

Supplementary Figure 1 The status of circulating immune cells between low-risk and high-risk groups.
