# Corrigendum: Construction and validation of a robust prognostic model based on immune features in sepsis

**DOI:** 10.3389/fimmu.2023.1146121

**Published:** 2023-03-06

**Authors:** Yongxin Zheng, Baiyun Liu, Xiumei Deng, Yubiao Chen, Yongbo Huang, Yu Zhang, Yonghao Xu, Ling Sang, Xiaoqing Liu, Yimin Li

**Affiliations:** ^1^ State Key Laboratory of Respiratory Diseases, National Clinical Research Center for Respiratory Disease, Guangzhou Institute of Respiratory Health, Department of Critical Care Medicine, The First Affiliated Hospital of Guangzhou Medical University, Guangzhou, China; ^2^ The First Affiliated Hospital, Guangzhou Medical University, Guangzhou, China

**Keywords:** sepsis, immune, prognostic model, 28-day mortality, immunosuppression

In the published article, there was an error. The ‘pheatmap’ R package should be corrected to ‘ggplot2’ R package; false discovery rate (FDR) should be corrected to P-value.

A correction has been made to **Materials and Methods, Differential expression analysis in sepsis.** This sentence previously stated:

“All the genes in GSE65682 were differentially analyzed by using limma R packages (http://www.bioconductor.org/packages/release/bioc/html/limma.html) (11). The parameter for DEGs screened was│Log2Foldchange│≥0.5 and false discovery rate (FDR) < 0.05. The Volcano plots were drawn by ‘pheatmap’ R package. Then, the IRGs that were overlapping with DEGs were identified as DEIRGs. Similarly, DETFs were obtained by matching TFs with DEGs.”

The corrected sentence appears below:

“All the genes in GSE65682 were differentially analyzed by using limma R packages (http://www.bioconductor.org/packages/release/bioc/html/limma.html) (11). The parameter for DEGs screened was│Log2Foldchange│≥0.5 and P-value <0.05. The Volcano plots were drawn by ‘ggplot2’ R package. Then, the IRGs that were overlapping with DEGs were identified as DEIRGs. Similarly, DETFs were obtained by matching TFs with DEGs.”

In the published article, there was an error. The ‘survival R’ package should be corrected to ‘survival’ R package.

A further correction has been made to **Materials and Methods, Construction of the prognostic prediction model in sepsis and development of nomogram**, paragraph 1. This section previously stated:

“Based on the univariate Cox regression analysis, prognostic DEIRGs were recognized as the biomarkers for multivariate Cox regression analysis. According to the median risk score value, conducted between low-risk and high-risk groups by using ‘survival R’ package. To evaluate the sensitivity and specificity of the prediction model, the receiver operating characteristics (ROC) curve was calculated using the ‘survivalROC’ package. The area under the ROC curve (AUC) was used to evaluate the prognostic model: 0.5-0.7 (moderate), 0.7-0.8 (better), and >0.9 (excellent).”

The corrected sentence appears below:

“Based on the univariate Cox regression analysis, prognostic DEIRGs were recognized as the biomarkers for multivariate Cox regression analysis. According to the median risk score value, conducted between low-risk and high-risk groups by using ‘survival’ R package. To evaluate the sensitivity and specificity of the prediction model, the receiver operating characteristics (ROC) curve was calculated using the ‘survivalROC’ package. The area under the ROC curve (AUC) was used to evaluate the prognostic model: 0.5-0.7 (moderate), 0.7-0.8 (better), and >0.9 (excellent).”

In the published article, there was an error in **Table 1** as published. The name of dataset “GSE63062” was wrong, and it should be changed to “GSE63042”. The corrected **Table 1** and its caption “Basic information of the datasets included in this study.” appear below.

**Table 1:** Basic information of the datasets included in this study.

Accession	Study population	Sample type	Country	Timing of gene expression profiling	Mortality/Total patients
GSE65682	Patient diagnoses sepsis due to cap, hap and non-infectious control.	Blood	Netherlands and England	On ICU admission	114/802
GSE63042	Patients with SIRS or sepsis	Blood	America	The day of enrollment upon presentation to the ED.	28/129
GSE95233	Patients with septic shock and healthy volunteers	Blood	France	Day 1 of ICU admission	34/124
GSE106878	septic shock patients from the CORTICUS-trial	Circulating leukocytes	International	Before hydrocortisone application	26/94
E-MTAB-4451	Patients with severe sepsis due to CAP	Circulating leukocytes	England	On ICU admission	52/106
E-MTAB-5273	Patients with sepsis due to CAP or faecal peritonitis.	Circulating leukocytes	England	First day of ICU stay	43/221
E-MTAB-5274	Patients with sepsis due to CAP or faecal peritonitis.	Circulating leukocytes	England	First day of ICU stay	14/106

The authors apologize for these errors and state that they do not change the scientific conclusions of the article in any way. The original article has been updated.

