# Supplementary figures and images for: Construction and validation of a robust prognostic model based on immune features in sepsis
Source: Front Immunol. 2022 Dec 2;13:994295. doi: 10.3389/fimmu.2022.994295 (PMC9756843; doi:10.3389/fimmu.2022.994295)

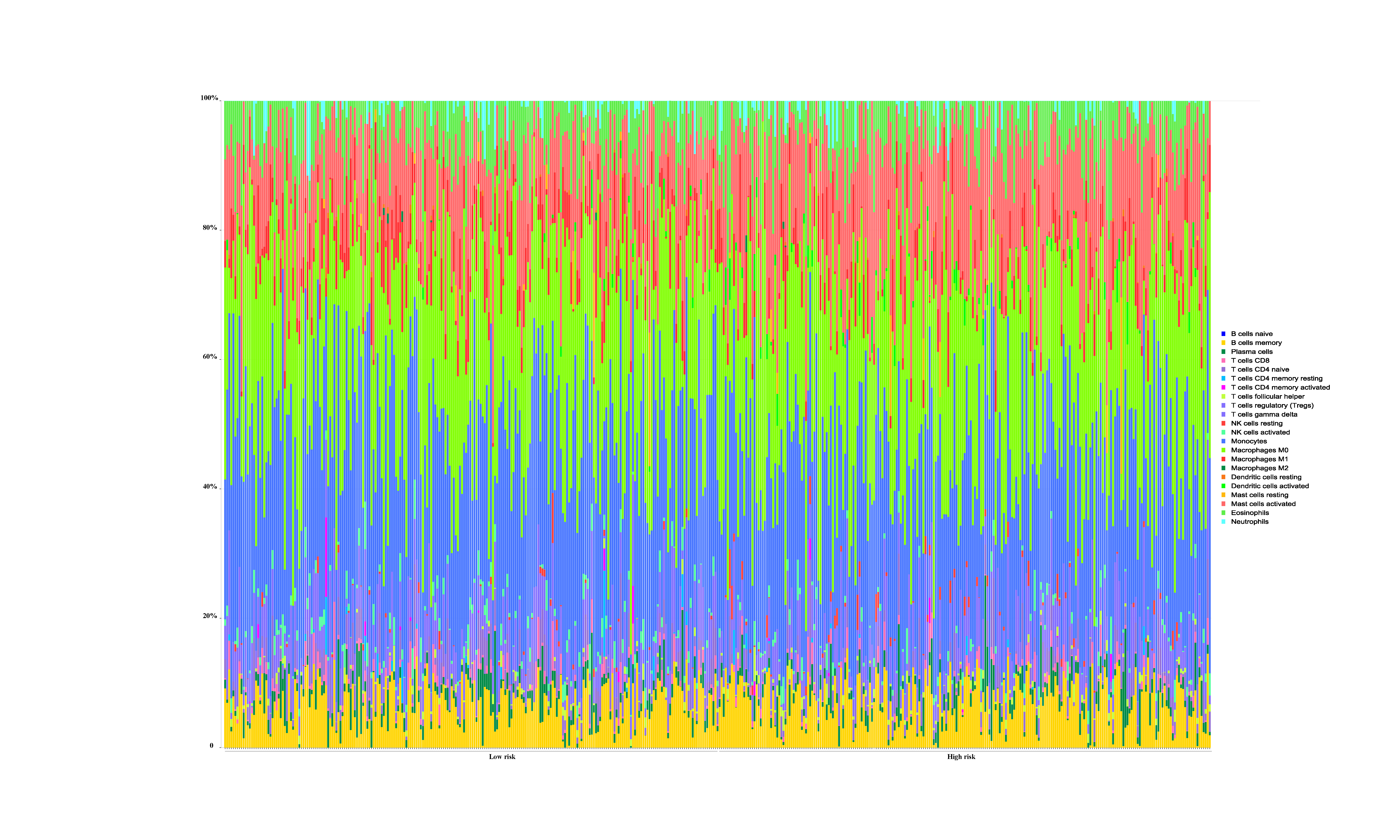

Supplement: Supplementary Figure 1 — The status of circulating immune cells between low-risk and high-risk groups. [file Image_1.tif]
