# Supplementary material for: Construction and validation of a robust prognostic model based on immune features in sepsis
Source: Front Immunol. 2022 Dec 2;13:994295. doi: 10.3389/fimmu.2022.994295 (PMC9756843; doi:10.3389/fimmu.2022.994295)
Supplement: Supplementary file 6 [file Table_5.docx]

Table S5. Univariate Cox regression analyses to identify risk DEIRGs in sepsis.

| ID | HR | HR.95L | HR.95H | pvalue |
| --- | --- | --- | --- | --- |
| ADRB2 | 0.651116 | 0.502486 | 0.84371 | 0.001173 |
| APOBEC3 | 0.724608 | 0.568961 | 0.922834 | 0.009032 |
| ARRB1 | 0.469355 | 0.321782 | 0.684607 | 8.59E-05 |
| AZU1 | 1.192768 | 1.045517 | 1.360757 | 0.00874 |
| CCL5 | 0.753263 | 0.643322 | 0.881991 | 0.000432 |
| CCR3 | 0.76803 | 0.629499 | 0.937046 | 0.009304 |
| CD1D | 0.751624 | 0.618755 | 0.913024 | 0.004018 |
| CD244 | 0.466143 | 0.311155 | 0.698332 | 0.000215 |
| CD3D | 0.762991 | 0.637937 | 0.91256 | 0.003058 |
| CD3E | 0.571117 | 0.384403 | 0.848523 | 0.005552 |
| CD3G | 0.757865 | 0.635758 | 0.903426 | 0.001982 |
| CD74 | 0.66784 | 0.541447 | 0.823738 | 0.000162 |
| CETP | 0.637686 | 0.460852 | 0.882374 | 0.006624 |
| CTSG | 1.167632 | 1.074096 | 1.269314 | 0.000275 |
| CTSS | 0.506506 | 0.368405 | 0.696377 | 2.82E-05 |
| CX3CR1 | 0.67905 | 0.59696 | 0.772429 | 3.91E-09 |
| CYSLTR1 | 0.610722 | 0.454509 | 0.820625 | 0.00107 |
| DDX58 | 0.809515 | 0.691165 | 0.94813 | 0.008781 |
| DEFA4 | 1.168014 | 1.077377 | 1.266275 | 0.000164 |
| ELANE | 1.184469 | 1.084029 | 1.294216 | 0.000181 |
| FCGR3A | 0.70257 | 0.578675 | 0.852991 | 0.000362 |
| FLT3LG | 0.528841 | 0.335412 | 0.833821 | 0.006102 |
| FURIN | 1.624832 | 1.175816 | 2.245316 | 0.003267 |
| FYN | 0.751148 | 0.604465 | 0.933425 | 0.009838 |
| GNLY | 0.807224 | 0.69322 | 0.939976 | 0.005837 |
| HCK | 0.645407 | 0.48208 | 0.864068 | 0.003267 |
| HLA-DMA | 0.761237 | 0.642859 | 0.901414 | 0.001559 |
| HLA-DMB | 0.695654 | 0.536309 | 0.902343 | 0.006253 |
| HLA-DPA | 0.742686 | 0.620555 | 0.888852 | 0.001173 |
| HLA-DPB | 0.704199 | 0.561501 | 0.883162 | 0.002403 |
| HLA-DRA | 0.739734 | 0.61596 | 0.88838 | 0.001252 |
| HLA-DRB | 0.740227 | 0.627734 | 0.872881 | 0.000348 |
| HLA-F | 0.59036 | 0.437125 | 0.797311 | 0.000588 |
| IL16 | 0.558237 | 0.376004 | 0.828792 | 0.003836 |
| IL17RA | 0.673914 | 0.514608 | 0.882536 | 0.00413 |
| IL1R2 | 1.268667 | 1.07433 | 1.498157 | 0.00503 |
| IL23A | 0.7468 | 0.60782 | 0.917559 | 0.005454 |
| IL27RA | 0.570584 | 0.411853 | 0.790492 | 0.000742 |
| ID | HR | HR.95L | HR.95H | pvalue |
| IL32 | 0.752919 | 0.61744 | 0.918126 | 0.005048 |
| IL4R | 0.683581 | 0.518515 | 0.901195 | 0.006981 |
| IRF1 | 0.685987 | 0.550949 | 0.854122 | 0.000752 |
| ISG20L2 | 0.520819 | 0.377221 | 0.719082 | 7.38E-05 |
| ITGAL | 0.427682 | 0.303604 | 0.602469 | 1.18E-06 |
| JAK1 | 0.583538 | 0.412057 | 0.826383 | 0.002412 |
| KLRK1 | 0.750329 | 0.614148 | 0.916706 | 0.004938 |
| LCK | 0.662579 | 0.530015 | 0.828299 | 0.000302 |
| LTB | 0.560744 | 0.437349 | 0.718955 | 5.07E-06 |
| MPO | 1.196641 | 1.078461 | 1.327771 | 0.000715 |
| NFATC2 | 0.74108 | 0.592824 | 0.926412 | 0.00851 |
| NFATC3 | 0.697312 | 0.538448 | 0.903048 | 0.006275 |
| OPRL1 | 0.557454 | 0.421517 | 0.737228 | 4.17E-05 |
| PAK1 | 0.56542 | 0.44093 | 0.725057 | 6.99E-06 |
| PLXNC1 | 0.689825 | 0.556965 | 0.854377 | 0.000669 |
| PRKCQ | 0.589058 | 0.399664 | 0.868204 | 0.007494 |
| PROK2 | 0.768033 | 0.648319 | 0.909852 | 0.002268 |
| PSME1 | 0.602752 | 0.422798 | 0.859299 | 0.005141 |
| PTX3 | 1.285224 | 1.103114 | 1.497398 | 0.001287 |
| RASGRP1 | 0.634367 | 0.449626 | 0.895015 | 0.009555 |
| RNASE3 | 1.202126 | 1.06447 | 1.357583 | 0.003009 |
| TAP2 | 0.717311 | 0.587379 | 0.875986 | 0.00112 |
| TAPBPL | 0.599999 | 0.429421 | 0.838335 | 0.00276 |
| TFRC | 1.396702 | 1.211104 | 1.610741 | 4.37E-06 |
| THBS1 | 1.301095 | 1.101942 | 1.536242 | 0.001902 |
| TMSB10 | 0.477901 | 0.328347 | 0.695573 | 0.000115 |
| TNFRSF10 | 0.742635 | 0.598585 | 0.921351 | 0.006841 |
| TNFRSF25 | 0.538868 | 0.337923 | 0.859305 | 0.009409 |
| TNFSF12 | 0.298961 | 0.188623 | 0.473843 | 2.77E-07 |
| TRBC1 | 0.808455 | 0.707963 | 0.923212 | 0.001691 |
| TRBV9 | 0.696222 | 0.532681 | 0.909973 | 0.008036 |

HR: Hazard ratio; L: low; H: high
