# Supplementary material for: Construction and validation of a robust prognostic model based on immune features in sepsis
Source: Front Immunol. 2022 Dec 2;13:994295. doi: 10.3389/fimmu.2022.994295 (PMC9756843; doi:10.3389/fimmu.2022.994295)
Supplement: Supplementary file 7 [file Table_6.docx]

Table S6. The correlation and regulation between TFs and DEIRGs.

| TFs | DEIRGs | cor | pvalue | Regulation |
| --- | --- | --- | --- | --- |
| BCL11B | CD244 | 0.577299 | 6.47E-44 | postive |
| BCL11B | CD3D | 0.883499 | 3.28E-159 | postive |
| BCL11B | CD3E | 0.757484 | 2.24E-90 | postive |
| BCL11B | CD3G | 0.832336 | 2.50E-124 | postive |
| BCL11B | CD74 | 0.502458 | 5.25E-32 | postive |
| BCL11B | CX3CR1 | 0.557482 | 1.83E-40 | postive |
| BCL11B | FLT3LG | 0.719399 | 1.63E-77 | postive |
| BCL11B | FYN | 0.638851 | 2.70E-56 | postive |
| BCL11B | GNLY | 0.501776 | 6.54E-32 | postive |
| BCL11B | HLA-DPA | 0.550576 | 2.57E-39 | postive |
| BCL11B | HLA-DPB | 0.563148 | 1.99E-41 | postive |
| BCL11B | IL1R2 | -0.61045 | 3.03E-50 | negative |
| BCL11B | IL23A | 0.830752 | 1.92E-123 | postive |
| BCL11B | IL32 | 0.774338 | 7.38E-97 | postive |
| BCL11B | KLRK1 | 0.677195 | 1.50E-65 | postive |
| BCL11B | LCK | 0.875415 | 1.05E-152 | postive |
| BCL11B | NFATC2 | 0.751144 | 4.49E-88 | postive |
| BCL11B | NFATC3 | 0.563955 | 1.45E-41 | postive |
| BCL11B | PRKCQ | 0.816964 | 4.16E-116 | postive |
| BCL11B | RASGRP1 | 0.877587 | 2.09E-154 | postive |
| BCL11B | TNFRSF25 | 0.715743 | 2.16E-76 | postive |
| BCL11B | TRBC1 | 0.838415 | 8.17E-128 | postive |
| CEBPE | AZU1 | 0.681593 | 1.06E-66 | postive |
| CEBPE | CTSG | 0.6894 | 8.46E-69 | postive |
| CEBPE | DEFA4 | 0.650134 | 7.03E-59 | postive |
| CEBPE | ELANE | 0.709975 | 1.17E-74 | postive |
| CEBPE | MPO | 0.762953 | 2.02E-92 | postive |
| CEBPE | PTX3 | 0.608958 | 6.06E-50 | postive |
| CEBPE | RNASE3 | 0.744675 | 8.52E-86 | postive |
| KLF1 | FURIN | 0.588206 | 6.44E-46 | postive |
| KLF1 | HCK | -0.53796 | 2.77E-37 | negative |
| KLF1 | IL17RA | -0.51604 | 5.94E-34 | negative |
| KLF1 | ISG20L2 | -0.55459 | 5.57E-40 | negative |
| KLF10 | CD1D | 0.542257 | 5.75E-38 | postive |
| MXI1 | FURIN | 0.640494 | 1.15E-56 | postive |
| MXI1 | HCK | -0.59277 | 8.89E-47 | negative |
| MXI1 | IL17RA | -0.59326 | 7.16E-47 | negative |
| MXI1 | ISG20L2 | -0.61844 | 6.94E-52 | negative |
| TF | DEIRGs | cor | pvalue | Regulation |
| MYC | CD3D | 0.52461 | 3.16E-35 | postive |
| MYC | CD74 | 0.556223 | 2.97E-40 | postive |
| MYC | FYN | 0.507136 | 1.15E-32 | postive |
| MYC | HLA-DPA | 0.510978 | 3.23E-33 | postive |
| MYC | HLA-DRB | 0.500563 | 9.65E-32 | postive |
| MYC | LCK | 0.505338 | 2.06E-32 | postive |
| MYC | PRKCQ | 0.515457 | 7.23E-34 | postive |
| MYC | RASGRP1 | 0.564756 | 1.05E-41 | postive |
| POLB | CD74 | 0.510926 | 3.29E-33 | postive |
| POLB | CTSS | 0.627506 | 8.38E-54 | postive |
| POLB | CYSLTR1 | 0.542862 | 4.60E-38 | postive |
| POLB | DDX58 | 0.724515 | 4.07E-79 | postive |
| POLB | HLA-DMB | 0.505224 | 2.14E-32 | postive |
| POLB | HLA-F | 0.624223 | 4.22E-53 | postive |
| POLB | IRF1 | 0.770513 | 2.44E-95 | postive |
| POLB | PSME1 | 0.743821 | 1.68E-85 | postive |
| POLB | TAP2 | 0.79127 | 5.80E-104 | postive |
| POLB | TMSB10 | 0.509357 | 5.53E-33 | postive |
| RUNX2 | CTSS | 0.605744 | 2.66E-49 | postive |
| RUNX2 | JAK1 | 0.550477 | 2.67E-39 | postive |
| RUNX2 | TNFRSF10 | 0.549101 | 4.49E-39 | postive |
| STAT1 | CYSLTR1 | 0.538554 | 2.23E-37 | postive |
| STAT1 | DDX58 | 0.873175 | 5.56E-151 | postive |
| STAT1 | HLA-F | 0.666077 | 1.00E-62 | postive |
| STAT1 | IRF1 | 0.815428 | 2.50E-115 | postive |
| STAT1 | PSME1 | 0.792483 | 1.70E-104 | postive |
| STAT1 | TAP2 | 0.879951 | 2.68E-156 | postive |
| TFDP1 | FURIN | 0.546488 | 1.20E-38 | postive |
| TFDP1 | IL17RA | -0.56386 | 1.50E-41 | negative |
| TFDP1 | ISG20L2 | -0.60954 | 4.62E-50 | negative |
| TFDP1 | ITGAL | -0.56756 | 3.44E-42 | negative |

TFs: transcription factors; DEIRGs: differentially expressed immune-related genes.
