# Supplementary material for: Construction and validation of a robust prognostic model based on immune features in sepsis
Source: Front Immunol. 2022 Dec 2;13:994295. doi: 10.3389/fimmu.2022.994295 (PMC9756843; doi:10.3389/fimmu.2022.994295)
Supplement: Supplementary file 8 [file Table_7.docx]

Table S7. The riskScore in sepsis.

| id | futime (day) | fustat | riskScore | risk |
| --- | --- | --- | --- | --- |
| GSM1602802 | 28 | 0 | 0.426268166 | low |
| GSM1602803 | 28 | 0 | 0.081516381 | low |
| GSM1602805 | 28 | 0 | 1.060330888 | high |
| GSM1602810 | 28 | 0 | 0.192071992 | low |
| GSM1602811 | 28 | 0 | 0.316214015 | low |
| GSM1602812 | 28 | 0 | 1.844347262 | high |
| GSM1602813 | 4 | 1 | 0.97436641 | low |
| GSM1602815 | 28 | 0 | 5.821093456 | high |
| GSM1602816 | 28 | 0 | 0.431491337 | low |
| GSM1602818 | 28 | 0 | 2.636719826 | high |
| GSM1602819 | 28 | 0 | 0.494904034 | low |
| GSM1602820 | 28 | 0 | 0.533063368 | low |
| GSM1602823 | 17 | 1 | 1.971813985 | high |
| GSM1602824 | 28 | 0 | 3.988244126 | high |
| GSM1602825 | 28 | 0 | 0.994033494 | low |
| GSM1602826 | 28 | 0 | 0.08008233 | low |
| GSM1602827 | 28 | 0 | 0.523498367 | low |
| GSM1602829 | 28 | 0 | 0.421003668 | low |
| GSM1602830 | 28 | 0 | 0.068875417 | low |
| GSM1602831 | 28 | 0 | 0.49219441 | low |
| GSM1602832 | 28 | 0 | 0.910567992 | low |
| GSM1602834 | 28 | 0 | 0.51220857 | low |
| GSM1602836 | 28 | 0 | 0.117560481 | low |
| GSM1602837 | 28 | 0 | 0.067197542 | low |
| GSM1602838 | 28 | 0 | 0.810852758 | low |
| GSM1602839 | 12 | 1 | 3.3488332 | high |
| GSM1602840 | 28 | 0 | 0.09668965 | low |
| GSM1602841 | 28 | 0 | 0.605864538 | low |
| GSM1602842 | 28 | 0 | 0.542626899 | low |
| GSM1602843 | 28 | 0 | 1.373868695 | high |
| GSM1602844 | 7 | 1 | 7.81426628 | high |
| GSM1602845 | 28 | 0 | 0.276000216 | low |
| GSM1602846 | 28 | 0 | 0.087196427 | low |
| GSM1602847 | 28 | 0 | 1.247778731 | high |
| GSM1602848 | 28 | 0 | 1.418624714 | high |
| GSM1602849 | 11 | 1 | 0.578028968 | low |
| GSM1602850 | 28 | 0 | 0.345997298 | low |
| GSM1602851 | 14 | 1 | 0.893728409 | low |
| id | futime (day) | fustat | riskScore | risk |
| GSM1602852 | 28 | 0 | 3.245521906 | high |
| GSM1602853 | 28 | 0 | 0.862541894 | low |
| GSM1602855 | 28 | 0 | 0.624465362 | low |
| GSM1602856 | 28 | 0 | 1.38508946 | high |
| GSM1602857 | 28 | 0 | 3.515720945 | high |
| GSM1602858 | 15 | 1 | 3.872361019 | high |
| GSM1602859 | 28 | 0 | 0.515503307 | low |
| GSM1602860 | 28 | 0 | 0.447645395 | low |
| GSM1602861 | 28 | 0 | 1.275273483 | high |
| GSM1602863 | 1 | 1 | 7.530811738 | high |
| GSM1602866 | 28 | 0 | 0.079830224 | low |
| GSM1602868 | 2 | 1 | 21.6018703 | high |
| GSM1602869 | 28 | 0 | 1.332413592 | high |
| GSM1602873 | 28 | 0 | 0.372628224 | low |
| GSM1602878 | 28 | 0 | 0.105398315 | low |
| GSM1602879 | 28 | 0 | 3.655236105 | high |
| GSM1602880 | 0 | 1 | 3.93850508 | high |
| GSM1602881 | 28 | 0 | 0.091420344 | low |
| GSM1602882 | 28 | 0 | 0.155466247 | low |
| GSM1602883 | 28 | 0 | 0.933546807 | low |
| GSM1602885 | 28 | 0 | 0.038110184 | low |
| GSM1602886 | 28 | 0 | 0.267076879 | low |
| GSM1602888 | 8 | 0 | 0.096026232 | low |
| GSM1602889 | 28 | 0 | 1.253833705 | high |
| GSM1602902 | 28 | 0 | 2.269214919 | high |
| GSM1602903 | 17 | 1 | 2.472481505 | high |
| GSM1602906 | 28 | 0 | 0.775342863 | low |
| GSM1602907 | 28 | 0 | 1.003674787 | high |
| GSM1602908 | 0 | 1 | 11.48550871 | high |
| GSM1602912 | 8 | 1 | 1.80983365 | high |
| GSM1602921 | 28 | 0 | 0.330821259 | low |
| GSM1602922 | 28 | 0 | 1.87454359 | high |
| GSM1602923 | 1 | 1 | 38.53985608 | high |
| GSM1602924 | 28 | 0 | 1.769637258 | high |
| GSM1602925 | 28 | 0 | 0.120044532 | low |
| GSM1602927 | 0 | 1 | 1.687034056 | high |
| GSM1602928 | 14 | 1 | 1.529341508 | high |
| GSM1602929 | 28 | 0 | 2.43216622 | high |
| GSM1602930 | 28 | 0 | 1.788961587 | high |
| id | futime (day) | fustat | riskScore | risk |
| GSM1602931 | 20 | 1 | 0.238430172 | low |
| GSM1602932 | 15 | 1 | 1.590715411 | high |
| GSM1602933 | 28 | 0 | 1.550937674 | high |
| GSM1602934 | 22 | 1 | 2.087010572 | high |
| GSM1602935 | 28 | 0 | 1.670470563 | high |
| GSM1602936 | 28 | 0 | 0.872658369 | low |
| GSM1602937 | 28 | 0 | 1.45913696 | high |
| GSM1602940 | 28 | 0 | 1.619391306 | high |
| GSM1602942 | 28 | 1 | 2.467106982 | high |
| GSM1602943 | 16 | 1 | 0.518316091 | low |
| GSM1602944 | 28 | 0 | 2.902814744 | high |
| GSM1602945 | 28 | 0 | 0.632573792 | low |
| GSM1602946 | 8 | 1 | 9.286306318 | high |
| GSM1602950 | 28 | 0 | 0.864817944 | low |
| GSM1602952 | 28 | 0 | 0.246594388 | low |
| GSM1602953 | 28 | 0 | 2.044506961 | high |
| GSM1602958 | 28 | 0 | 1.568341911 | high |
| GSM1602968 | 28 | 0 | 1.770469621 | high |
| GSM1602973 | 28 | 0 | 0.385702877 | low |
| GSM1602974 | 28 | 0 | 2.250228939 | high |
| GSM1602975 | 28 | 0 | 0.043145503 | low |
| GSM1602976 | 28 | 0 | 2.592226192 | high |
| GSM1691861 | 6 | 1 | 2.278181367 | high |
| GSM1691863 | 28 | 0 | 1.484286568 | high |
| GSM1691864 | 12 | 1 | 3.754885845 | high |
| GSM1691865 | 28 | 0 | 0.916873923 | low |
| GSM1691866 | 28 | 0 | 0.778188294 | low |
| GSM1691867 | 28 | 0 | 3.576147116 | high |
| GSM1691868 | 7 | 1 | 0.340081776 | low |
| GSM1691871 | 28 | 0 | 0.660888953 | low |
| GSM1691873 | 28 | 0 | 0.765620962 | low |
| GSM1691874 | 28 | 0 | 1.738975615 | high |
| GSM1691875 | 28 | 0 | 4.090269231 | high |
| GSM1691877 | 28 | 0 | 0.878448732 | low |
| GSM1691878 | 28 | 0 | 0.925957831 | low |
| GSM1691880 | 11 | 1 | 3.895319201 | high |
| GSM1691882 | 28 | 0 | 7.166668324 | high |
| GSM1691883 | 28 | 0 | 1.556876563 | high |
| GSM1691884 | 0 | 1 | 5.426986482 | high |
| id | futime (day) | fustat | riskScore | risk |
| GSM1691885 | 28 | 0 | 1.534717366 | high |
| GSM1691886 | 28 | 0 | 0.314301459 | low |
| GSM1691890 | 28 | 0 | 0.175799961 | low |
| GSM1691893 | 28 | 0 | 2.037631879 | high |
| GSM1691895 | 28 | 0 | 0.38812548 | low |
| GSM1691896 | 28 | 0 | 0.604828757 | low |
| GSM1691897 | 28 | 0 | 0.815532264 | low |
| GSM1691898 | 28 | 0 | 2.002091147 | high |
| GSM1691900 | 13 | 1 | 4.738378186 | high |
| GSM1691901 | 28 | 0 | 0.223961128 | low |
| GSM1691903 | 28 | 0 | 1.036110041 | high |
| GSM1691904 | 28 | 0 | 0.68247951 | low |
| GSM1691905 | 28 | 0 | 0.559864143 | low |
| GSM1691909 | 28 | 0 | 0.1964606 | low |
| GSM1691911 | 28 | 0 | 0.443112114 | low |
| GSM1691915 | 28 | 0 | 1.396083578 | high |
| GSM1691918 | 28 | 0 | 2.801775536 | high |
| GSM1691923 | 28 | 0 | 0.099072318 | low |
| GSM1691925 | 28 | 0 | 0.145334382 | low |
| GSM1691927 | 28 | 0 | 1.555070199 | high |
| GSM1691928 | 28 | 0 | 0.214143946 | low |
| GSM1691929 | 28 | 0 | 0.50726138 | low |
| GSM1691932 | 4 | 1 | 3.831555812 | high |
| GSM1691933 | 28 | 0 | 1.448716296 | high |
| GSM1691934 | 28 | 0 | 0.344130989 | low |
| GSM1691935 | 15 | 1 | 0.860033332 | low |
| GSM1691937 | 7 | 1 | 4.920198047 | high |
| GSM1691938 | 28 | 0 | 0.129183656 | low |
| GSM1691941 | 28 | 0 | 7.00712225 | high |
| GSM1691942 | 28 | 0 | 2.36566131 | high |
| GSM1691943 | 12 | 1 | 3.880012411 | high |
| GSM1691944 | 28 | 0 | 0.308955068 | low |
| GSM1691946 | 8 | 1 | 0.480110751 | low |
| GSM1691947 | 28 | 0 | 0.453081962 | low |
| GSM1691953 | 28 | 0 | 0.254942452 | low |
| GSM1691954 | 28 | 0 | 1.749441113 | high |
| GSM1691957 | 28 | 0 | 4.540168121 | high |
| GSM1691959 | 28 | 0 | 0.210646934 | low |
| GSM1691960 | 28 | 0 | 0.152099965 | low |
| id | futime (day) | fustat | riskScore | risk |
| GSM1691961 | 28 | 0 | 0.257289068 | low |
| GSM1691962 | 28 | 0 | 1.807246766 | high |
| GSM1691963 | 28 | 0 | 0.284488396 | low |
| GSM1691965 | 15 | 1 | 0.233246683 | low |
| GSM1691966 | 8 | 1 | 9.804525419 | high |
| GSM1691968 | 28 | 0 | 0.309673479 | low |
| GSM1691969 | 0 | 1 | 18.55648202 | high |
| GSM1691970 | 28 | 0 | 2.617991756 | high |
| GSM1691973 | 28 | 0 | 0.340618854 | low |
| GSM1691977 | 28 | 0 | 0.290299185 | low |
| GSM1691980 | 28 | 0 | 0.811620308 | low |
| GSM1691981 | 0 | 1 | 21.70468645 | high |
| GSM1691983 | 28 | 0 | 6.128542192 | high |
| GSM1691984 | 2 | 1 | 14.24476771 | high |
| GSM1691985 | 13 | 1 | 2.502990969 | high |
| GSM1691986 | 28 | 0 | 0.795174238 | low |
| GSM1691987 | 28 | 0 | 2.73108942 | high |
| GSM1691989 | 28 | 0 | 0.205348903 | low |
| GSM1691990 | 28 | 0 | 0.364638491 | low |
| GSM1691991 | 5 | 1 | 3.014341895 | high |
| GSM1691992 | 14 | 1 | 0.503457127 | low |
| GSM1691993 | 28 | 0 | 0.25548931 | low |
| GSM1691994 | 28 | 0 | 2.437946397 | high |
| GSM1691995 | 28 | 0 | 13.77228482 | high |
| GSM1691997 | 28 | 0 | 1.102656175 | high |
| GSM1692000 | 28 | 0 | 0.201313003 | low |
| GSM1692002 | 28 | 0 | 0.92198327 | low |
| GSM1692004 | 6 | 1 | 0.863199227 | low |
| GSM1692005 | 28 | 0 | 1.109428396 | high |
| GSM1692006 | 3 | 1 | 3.180646745 | high |
| GSM1692007 | 28 | 0 | 0.500312704 | low |
| GSM1692008 | 28 | 0 | 0.658606797 | low |
| GSM1692009 | 28 | 0 | 1.854408864 | high |
| GSM1692011 | 2 | 1 | 4.453150193 | high |
| GSM1692012 | 28 | 0 | 0.680515918 | low |
| GSM1692016 | 28 | 0 | 1.0031994 | low |
| GSM1692017 | 2 | 1 | 5.323893195 | high |
| GSM1692019 | 28 | 0 | 0.879554509 | low |
| GSM1692022 | 28 | 0 | 6.350959423 | high |
| id | futime (day) | fustat | riskScore | risk |
| GSM1692023 | 28 | 0 | 1.731092529 | high |
| GSM1692024 | 3 | 1 | 1.036989194 | high |
| GSM1692025 | 28 | 0 | 0.091987522 | low |
| GSM1692029 | 3 | 1 | 5.932190331 | high |
| GSM1692030 | 28 | 0 | 0.83758539 | low |
| GSM1692032 | 28 | 0 | 0.187349943 | low |
| GSM1692033 | 28 | 0 | 0.658851798 | low |
| GSM1692034 | 28 | 0 | 0.098466061 | low |
| GSM1692035 | 28 | 0 | 0.09944904 | low |
| GSM1692038 | 12 | 1 | 3.912448476 | high |
| GSM1692040 | 28 | 0 | 0.673703517 | low |
| GSM1692041 | 28 | 0 | 1.460352337 | high |
| GSM1692042 | 28 | 0 | 0.58236075 | low |
| GSM1692043 | 1 | 1 | 2.382956464 | high |
| GSM1692045 | 28 | 0 | 0.705173803 | low |
| GSM1692047 | 1 | 1 | 6.755876691 | high |
| GSM1692050 | 28 | 0 | 0.659334033 | low |
| GSM1692051 | 28 | 0 | 0.141256354 | low |
| GSM1692052 | 28 | 0 | 0.881651462 | low |
| GSM1692055 | 28 | 0 | 0.602222245 | low |
| GSM1692056 | 1 | 1 | 11.19364486 | high |
| GSM1692058 | 28 | 0 | 0.393436124 | low |
| GSM1692059 | 28 | 0 | 0.176779731 | low |
| GSM1692060 | 28 | 0 | 7.391531479 | high |
| GSM1692061 | 28 | 0 | 0.513793203 | low |
| GSM1692063 | 28 | 0 | 0.667361941 | low |
| GSM1692064 | 6 | 1 | 23.51692207 | high |
| GSM1692065 | 28 | 0 | 0.355189992 | low |
| GSM1692066 | 28 | 0 | 0.977892158 | low |
| GSM1692068 | 28 | 0 | 4.713112384 | high |
| GSM1692071 | 1 | 1 | 2.501532273 | high |
| GSM1692073 | 28 | 0 | 1.931616971 | high |
| GSM1692074 | 28 | 0 | 0.107726119 | low |
| GSM1692077 | 28 | 0 | 0.24208515 | low |
| GSM1692079 | 1 | 1 | 39.30224486 | high |
| GSM1692081 | 28 | 0 | 5.007905626 | high |
| GSM1692084 | 28 | 0 | 2.533116689 | high |
| GSM1692086 | 28 | 0 | 1.743316752 | high |
| GSM1692087 | 28 | 0 | 0.539499822 | low |
| id | futime (day) | fustat | riskScore | risk |
| GSM1692089 | 28 | 0 | 0.17850745 | low |
| GSM1692092 | 5 | 1 | 1.756092521 | high |
| GSM1692093 | 28 | 0 | 0.167792288 | low |
| GSM1692094 | 28 | 0 | 0.35314646 | low |
| GSM1692096 | 28 | 0 | 0.612347198 | low |
| GSM1692099 | 28 | 0 | 1.950995817 | high |
| GSM1692100 | 5 | 1 | 1.614882859 | high |
| GSM1692101 | 28 | 0 | 0.017410349 | low |
| GSM1692103 | 28 | 0 | 1.496453266 | high |
| GSM1692105 | 0 | 1 | 1.638367783 | high |
| GSM1692107 | 28 | 0 | 0.420921668 | low |
| GSM1692108 | 28 | 0 | 5.245514158 | high |
| GSM1692109 | 28 | 0 | 0.781253641 | low |
| GSM1692112 | 3 | 1 | 0.885515313 | low |
| GSM1692114 | 12 | 1 | 2.574137859 | high |
| GSM1692115 | 28 | 0 | 1.904526206 | high |
| GSM1692116 | 28 | 0 | 0.77721848 | low |
| GSM1692117 | 28 | 0 | 0.911527113 | low |
| GSM1692118 | 28 | 0 | 3.456266444 | high |
| GSM1692119 | 6 | 1 | 6.136393165 | high |
| GSM1692121 | 28 | 0 | 0.118238168 | low |
| GSM1692123 | 28 | 0 | 0.430358952 | low |
| GSM1692124 | 14 | 1 | 1.792136741 | high |
| GSM1692125 | 28 | 0 | 0.269828482 | low |
| GSM1692126 | 28 | 0 | 0.817396176 | low |
| GSM1692127 | 28 | 0 | 0.261331394 | low |
| GSM1692130 | 28 | 0 | 0.136354826 | low |
| GSM1692132 | 28 | 0 | 0.204173605 | low |
| GSM1692133 | 28 | 0 | 1.815618186 | high |
| GSM1692136 | 28 | 0 | 1.990801826 | high |
| GSM1692137 | 28 | 0 | 1.056051528 | high |
| GSM1692138 | 28 | 0 | 0.386920433 | low |
| GSM1692139 | 28 | 0 | 4.319734219 | high |
| GSM1692140 | 28 | 0 | 2.217744362 | high |
| GSM1692141 | 28 | 0 | 0.704580539 | low |
| GSM1692142 | 28 | 0 | 1.31414553 | high |
| GSM1692143 | 28 | 0 | 0.248953022 | low |
| GSM1692144 | 4 | 1 | 0.907809868 | low |
| GSM1692145 | 28 | 0 | 0.544300673 | low |
| id | futime (day) | fustat | riskScore | risk |
| GSM1692146 | 3 | 1 | 2.657282483 | high |
| GSM1692148 | 10 | 1 | 6.798495831 | high |
| GSM1692149 | 28 | 1 | 5.737964535 | high |
| GSM1692151 | 15 | 1 | 2.736107568 | high |
| GSM1692153 | 28 | 0 | 1.1569957 | high |
| GSM1692156 | 28 | 0 | 0.258412449 | low |
| GSM1692157 | 28 | 0 | 0.310446134 | low |
| GSM1692158 | 28 | 0 | 0.454932364 | low |
| GSM1692160 | 2 | 1 | 1.689903316 | high |
| GSM1692162 | 28 | 0 | 0.120297788 | low |
| GSM1692163 | 18 | 1 | 3.574025947 | high |
| GSM1692165 | 28 | 0 | 0.491134217 | low |
| GSM1692166 | 28 | 0 | 0.782543324 | low |
| GSM1692167 | 28 | 0 | 0.079609937 | low |
| GSM1692168 | 28 | 0 | 0.419064387 | low |
| GSM1692169 | 12 | 1 | 0.531237224 | low |
| GSM1692170 | 7 | 1 | 14.25857498 | high |
| GSM1692172 | 28 | 0 | 0.600540512 | low |
| GSM1692174 | 28 | 0 | 9.144830839 | high |
| GSM1692175 | 4 | 1 | 6.480454225 | high |
| GSM1692181 | 28 | 0 | 5.092623714 | high |
| GSM1692182 | 3 | 1 | 0.362289511 | low |
| GSM1692184 | 1 | 1 | 22.50714559 | high |
| GSM1692186 | 2 | 1 | 2.196065198 | high |
| GSM1692187 | 28 | 0 | 0.576953879 | low |
| GSM1692188 | 28 | 0 | 1.124784135 | high |
| GSM1692190 | 3 | 1 | 7.164280817 | high |
| GSM1692191 | 28 | 0 | 3.708317762 | high |
| GSM1692192 | 28 | 0 | 0.195868847 | low |
| GSM1692193 | 28 | 0 | 0.571465439 | low |
| GSM1692196 | 28 | 0 | 1.562834067 | high |
| GSM1692197 | 28 | 0 | 0.409293482 | low |
| GSM1692198 | 28 | 0 | 6.061711091 | high |
| GSM1692199 | 28 | 0 | 0.143518737 | low |
| GSM1692201 | 28 | 0 | 1.256942671 | high |
| GSM1692202 | 28 | 0 | 0.755137598 | low |
| GSM1692203 | 28 | 0 | 5.914661598 | high |
| GSM1692204 | 28 | 0 | 0.283565061 | low |
| GSM1692205 | 23 | 1 | 1.922777552 | high |
| id | futime (day) | fustat | riskScore | risk |
| GSM1692206 | 28 | 0 | 0.817442064 | low |
| GSM1692207 | 28 | 0 | 1.599952369 | high |
| GSM1692209 | 6 | 1 | 3.999030731 | high |
| GSM1692211 | 12 | 1 | 3.097893132 | high |
| GSM1692212 | 28 | 0 | 0.621306289 | low |
| GSM1692213 | 28 | 0 | 1.624777751 | high |
| GSM1692214 | 7 | 1 | 9.287629816 | high |
| GSM1692216 | 28 | 0 | 1.24111169 | high |
| GSM1692217 | 28 | 0 | 0.233442378 | low |
| GSM1692221 | 28 | 0 | 1.015930962 | high |
| GSM1692222 | 28 | 0 | 0.353074266 | low |
| GSM1692223 | 14 | 1 | 1.926094728 | high |
| GSM1692224 | 27 | 1 | 0.964671172 | low |
| GSM1692225 | 28 | 0 | 2.06140012 | high |
| GSM1692226 | 28 | 0 | 0.330317127 | low |
| GSM1692227 | 28 | 0 | 0.096065686 | low |
| GSM1692228 | 28 | 0 | 0.339789527 | low |
| GSM1692230 | 28 | 0 | 1.292234047 | high |
| GSM1692232 | 28 | 0 | 0.252199532 | low |
| GSM1692238 | 28 | 0 | 2.460303347 | high |
| GSM1692240 | 28 | 0 | 1.096430931 | high |
| GSM1692241 | 16 | 1 | 3.092923692 | high |
| GSM1692242 | 28 | 0 | 0.875127202 | low |
| GSM1692243 | 28 | 0 | 0.236051238 | low |
| GSM1692244 | 7 | 1 | 0.702219808 | low |
| GSM1692245 | 28 | 0 | 0.083335264 | low |
| GSM1692247 | 28 | 0 | 0.452504836 | low |
| GSM1692251 | 3 | 1 | 6.146237549 | high |
| GSM1692255 | 28 | 0 | 1.413680947 | high |
| GSM1692257 | 28 | 0 | 0.722350983 | low |
| GSM1692258 | 28 | 0 | 0.472885174 | low |
| GSM1692259 | 28 | 0 | 0.194540327 | low |
| GSM1692260 | 28 | 0 | 0.430631502 | low |
| GSM1692261 | 28 | 0 | 3.664327579 | high |
| GSM1692264 | 10 | 1 | 0.30260577 | low |
| GSM1692266 | 28 | 0 | 0.236391621 | low |
| GSM1692268 | 0 | 1 | 4.462804321 | high |
| GSM1692269 | 28 | 0 | 0.399536491 | low |
| GSM1692271 | 10 | 1 | 3.625665951 | high |
| id | futime (day) | fustat | riskScore | risk |
| GSM1692272 | 28 | 0 | 0.658278227 | low |
| GSM1692274 | 28 | 0 | 3.833875754 | high |
| GSM1692277 | 28 | 0 | 1.134459422 | high |
| GSM1692278 | 28 | 0 | 2.176639332 | high |
| GSM1692279 | 2 | 1 | 0.600979687 | low |
| GSM1692284 | 28 | 0 | 1.445757702 | high |
| GSM1692286 | 28 | 0 | 2.060489856 | high |
| GSM1692289 | 28 | 0 | 0.709757068 | low |
| GSM1692291 | 28 | 0 | 0.309838096 | low |
| GSM1692292 | 0 | 1 | 1.654334907 | high |
| GSM1692294 | 28 | 0 | 0.407408519 | low |
| GSM1692296 | 28 | 0 | 0.44886347 | low |
| GSM1692298 | 28 | 0 | 3.882892367 | high |
| GSM1692299 | 21 | 1 | 2.159878496 | high |
| GSM1692300 | 28 | 0 | 1.45088316 | high |
| GSM1692301 | 28 | 0 | 7.12523133 | high |
| GSM1692302 | 28 | 0 | 1.430593445 | high |
| GSM1692303 | 28 | 0 | 1.75109229 | high |
| GSM1692304 | 28 | 0 | 0.834644616 | low |
| GSM1692305 | 28 | 0 | 2.085873451 | high |
| GSM1692308 | 28 | 0 | 3.368065432 | high |
| GSM1692309 | 28 | 0 | 0.310404129 | low |
| GSM1692311 | 11 | 1 | 1.783991375 | high |
| GSM1692312 | 0 | 1 | 30.05525789 | high |
| GSM1692315 | 28 | 0 | 0.126741398 | low |
| GSM1692316 | 1 | 1 | 8.611802568 | high |
| GSM1692317 | 28 | 0 | 0.246726431 | low |
| GSM1692318 | 1 | 1 | 2.483226868 | high |
| GSM1692319 | 28 | 0 | 0.022010441 | low |
| GSM1692322 | 28 | 0 | 1.037637419 | high |
| GSM1692326 | 25 | 1 | 1.414660992 | high |
| GSM1692327 | 28 | 0 | 0.108215584 | low |
| GSM1692329 | 5 | 1 | 9.044656585 | high |
| GSM1692330 | 28 | 0 | 1.159495856 | high |
| GSM1692331 | 2 | 1 | 2.260039449 | high |
| GSM1692332 | 28 | 0 | 2.806382426 | high |
| GSM1692335 | 28 | 0 | 1.460393751 | high |
| GSM1692336 | 1 | 1 | 0.597990548 | low |
| GSM1692337 | 28 | 0 | 0.66705723 | low |
| id | futime (day) | fustat | riskScore | risk |
| GSM1692338 | 28 | 0 | 0.852668502 | low |
| GSM1692340 | 2 | 1 | 0.928257934 | low |
| GSM1692343 | 8 | 1 | 4.095701154 | high |
| GSM1692347 | 28 | 0 | 5.879598334 | high |
| GSM1692348 | 28 | 0 | 1.208777257 | high |
| GSM1692350 | 0 | 1 | 10.36067607 | high |
| GSM1692352 | 8 | 1 | 5.234032906 | high |
| GSM1692355 | 28 | 0 | 0.930577732 | low |
| GSM1692356 | 28 | 0 | 0.451080646 | low |
| GSM1692357 | 28 | 0 | 0.223860164 | low |
| GSM1692360 | 28 | 0 | 0.194878412 | low |
| GSM1692361 | 28 | 0 | 0.47150552 | low |
| GSM1692362 | 28 | 0 | 0.874078308 | low |
| GSM1692364 | 28 | 0 | 7.002305846 | high |
| GSM1692366 | 28 | 0 | 0.936654495 | low |
| GSM1692369 | 15 | 1 | 1.179834144 | high |
| GSM1692373 | 28 | 0 | 2.309289404 | high |
| GSM1692374 | 28 | 0 | 0.403948261 | low |
| GSM1692375 | 28 | 0 | 0.291688308 | low |
| GSM1692376 | 11 | 1 | 3.324624426 | high |
| GSM1692377 | 2 | 1 | 0.457828836 | low |
| GSM1692379 | 5 | 0 | 2.172330308 | high |
| GSM1692381 | 28 | 0 | 2.077098873 | high |
| GSM1692384 | 28 | 0 | 1.405346536 | high |
| GSM1692386 | 28 | 0 | 0.159819434 | low |
| GSM1692387 | 1 | 1 | 1.441311691 | high |
| GSM1692388 | 18 | 1 | 2.096819618 | high |
| GSM1692389 | 19 | 1 | 0.132921839 | low |
| GSM1692391 | 28 | 0 | 2.97995016 | high |
| GSM1692392 | 28 | 0 | 0.361753782 | low |
| GSM1692393 | 28 | 0 | 0.77390922 | low |
| GSM1692394 | 1 | 1 | 8.912794997 | high |
| GSM1692395 | 28 | 0 | 1.049408905 | high |
| GSM1692397 | 28 | 0 | 0.482843644 | low |
| GSM1692399 | 28 | 0 | 1.447365358 | high |
| GSM1692400 | 28 | 0 | 0.68019314 | low |
| GSM1692404 | 28 | 0 | 0.046873081 | low |
| GSM1692406 | 28 | 0 | 0.201561303 | low |
| GSM1692407 | 28 | 0 | 0.134000204 | low |
| id | futime (day) | fustat | riskScore | risk |
| GSM1692408 | 28 | 0 | 0.349429096 | low |
| GSM1692410 | 28 | 0 | 1.43176814 | high |
| GSM1692412 | 28 | 0 | 3.312654577 | high |
| GSM1692413 | 28 | 0 | 0.778131005 | low |
| GSM1692414 | 28 | 0 | 1.851478174 | high |
| GSM1692415 | 28 | 0 | 0.105846222 | low |
| GSM1692416 | 28 | 0 | 1.145956955 | high |
| GSM1692417 | 28 | 0 | 1.063259057 | high |
| GSM1692418 | 28 | 0 | 0.710959856 | low |
| GSM1692420 | 28 | 0 | 1.181906402 | high |
| GSM1692421 | 28 | 0 | 0.803069013 | low |
| GSM1692422 | 28 | 0 | 1.644594487 | high |
| GSM1692423 | 28 | 0 | 0.209881434 | low |
| GSM1692424 | 28 | 0 | 0.659372373 | low |
| GSM1692425 | 28 | 0 | 0.942356664 | low |
| GSM1692427 | 28 | 0 | 2.422970385 | high |
| GSM1692428 | 28 | 0 | 1.27709873 | high |
| GSM1692429 | 28 | 0 | 1.393501045 | high |
| GSM1692430 | 28 | 0 | 1.870509331 | high |
| GSM1692436 | 28 | 0 | 0.298805028 | low |
| GSM1692437 | 28 | 0 | 0.838206673 | low |
| GSM1692438 | 28 | 0 | 0.669273624 | low |
| GSM1692439 | 28 | 0 | 2.311898494 | high |
| GSM1692440 | 28 | 0 | 0.829756027 | low |
| GSM1692441 | 28 | 0 | 0.888017312 | low |
| GSM1692443 | 8 | 1 | 1.189114435 | high |
| GSM1692445 | 28 | 0 | 1.825549235 | high |
| GSM1692446 | 28 | 0 | 0.563670565 | low |
| GSM1692448 | 28 | 0 | 0.477391802 | low |
| GSM1692452 | 28 | 0 | 3.615862625 | high |
| GSM1692453 | 28 | 0 | 4.201499217 | high |
| GSM1692454 | 17 | 1 | 2.473340758 | high |
| GSM1692455 | 28 | 0 | 0.319992723 | low |
| GSM1692458 | 28 | 0 | 2.837379439 | high |
| GSM1692459 | 20 | 1 | 2.068750383 | high |
| GSM1692461 | 28 | 0 | 1.540064735 | high |
| GSM1692465 | 28 | 0 | 2.719360547 | high |
| GSM1692466 | 28 | 0 | 0.149204133 | low |
| GSM1692467 | 1 | 1 | 18.90270512 | high |
| id | futime (day) | fustat | riskScore | risk |
| GSM1692478 | 13 | 1 | 4.710016254 | high |
| GSM1692482 | 28 | 0 | 1.18691523 | high |
| GSM1692484 | 28 | 0 | 2.18059931 | high |
| GSM1692488 | 28 | 0 | 1.793510975 | high |
| GSM1692489 | 28 | 0 | 0.808125002 | low |
| GSM1692491 | 28 | 0 | 1.004655724 | high |
| GSM1692492 | 28 | 0 | 0.43231795 | low |
| GSM1692493 | 28 | 0 | 1.320493547 | high |
| GSM1692494 | 28 | 0 | 0.624901408 | low |
| GSM1692498 | 28 | 0 | 1.968120821 | high |
| GSM1692499 | 28 | 0 | 5.844299235 | high |
| GSM1692501 | 28 | 0 | 0.830883202 | low |

**The formulas was used to construct survival riskscore value.**

survival riskscore value = (-0.693479) × ADRB2 expression + (-0.394755) × CD1D expression + 0.7029737 × CD74 expression + (-1.176492) × CETP expression + 0.3804079 × ELANE expression + 0.4151731 × FYN expression + (-0.244232) × GNLY expression + (-0.761003) × HLA-DRA expression + 1.0580916 × IL16 expression + (-0.54816) × IL17RA expression + 0.2860863 × IL1R2 expression + (-0.651796) × LTB expression + (-0.615383) × MPO expression + (-0.488971) × PLXNC1 expression + 0.7359292 × PSME1 expression + (-0.386602) × TAP2 expression + 0.1729455 × TFRC expression + 0.3465734 × THBS1 expression + 0.7411038 × TNFRSF10B expression + (-0.898271) × TNFSF12 expression + (-0.336649) × TRBV9 expression + 0.1906303 × DEFA4 expression.
