# Supplementary material for: Construction and validation of a robust prognostic model based on immune features in sepsis
Source: Front Immunol. 2022 Dec 2;13:994295. doi: 10.3389/fimmu.2022.994295 (PMC9756843; doi:10.3389/fimmu.2022.994295)
Supplement: Supplementary file 9 [file Table_8.docx]

Table S8. The 28-day mortality in high risk and low risk.

| risk=high | | | | | | |
| --- | --- | --- | --- | --- | --- | --- |
| time (days) | n.risk | n.event | survival | std.err | lower 95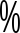 CI | upper 95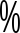 CI |
| 0 | 239 | 11 | 0.954 | 0.0136 | 0.928 | 0.981 |
| 1 | 228 | 13 | 0.9 | 0.0194 | 0.862 | 0.939 |
| 2 | 215 | 7 | 0.87 | 0.0217 | 0.829 | 0.914 |
| 3 | 208 | 6 | 0.845 | 0.0234 | 0.801 | 0.892 |
| 4 | 202 | 2 | 0.837 | 0.0239 | 0.791 | 0.885 |
| 5 | 200 | 4 | 0.82 | 0.0248 | 0.773 | 0.87 |
| 6 | 195 | 4 | 0.803 | 0.0257 | 0.754 | 0.855 |
| 7 | 191 | 4 | 0.786 | 0.0265 | 0.736 | 0.84 |
| 8 | 187 | 6 | 0.761 | 0.0276 | 0.709 | 0.817 |
| 10 | 181 | 2 | 0.753 | 0.0279 | 0.7 | 0.81 |
| 11 | 179 | 3 | 0.74 | 0.0284 | 0.687 | 0.798 |
| 12 | 176 | 6 | 0.715 | 0.0292 | 0.66 | 0.775 |
| 13 | 170 | 3 | 0.702 | 0.0296 | 0.647 | 0.763 |
| 14 | 167 | 3 | 0.69 | 0.03 | 0.633 | 0.751 |
| 15 | 164 | 4 | 0.673 | 0.0304 | 0.616 | 0.735 |
| 16 | 160 | 1 | 0.669 | 0.0305 | 0.612 | 0.731 |
| 17 | 159 | 3 | 0.656 | 0.0308 | 0.598 | 0.719 |
| 18 | 156 | 2 | 0.648 | 0.0309 | 0.59 | 0.711 |
| 20 | 154 | 1 | 0.643 | 0.031 | 0.585 | 0.707 |
| 21 | 153 | 1 | 0.639 | 0.0311 | 0.581 | 0.703 |
| 22 | 152 | 1 | 0.635 | 0.0312 | 0.577 | 0.699 |
| 23 | 151 | 1 | 0.631 | 0.0313 | 0.572 | 0.695 |
| 25 | 150 | 1 | 0.627 | 0.0313 | 0.568 | 0.691 |
| 28 | 149 | 2 | 0.618 | 0.0315 | 0.56 | 0.683 |

| risk=low | | | | | | |
| --- | --- | --- | --- | --- | --- | --- |
| Time (days) | n.risk | n.event | survival | std.err | lower 95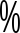 CI | upper 95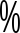 CI |
| 1 | 240 | 1 | 0.996 | 0.00416 | 0.988 | 1 |
| 2 | 239 | 3 | 0.983 | 0.00826 | 0.967 | 1 |
| 3 | 236 | 2 | 0.975 | 0.01008 | 0.955 | 0.995 |
| 4 | 234 | 2 | 0.967 | 0.01159 | 0.944 | 0.99 |
| 6 | 232 | 1 | 0.963 | 0.01226 | 0.939 | 0.987 |
| 7 | 231 | 2 | 0.954 | 0.0135 | 0.928 | 0.981 |
| 8 | 229 | 1 | 0.95 | 0.01407 | 0.923 | 0.978 |
| 10 | 227 | 1 | 0.946 | 0.01462 | 0.918 | 0.975 |
| 11 | 226 | 1 | 0.942 | 0.01514 | 0.912 | 0.972 |
| 12 | 225 | 1 | 0.937 | 0.01564 | 0.907 | 0.969 |
| risk=low | | | | | | |
| Time (days) | n.risk | n.event | survival | std.err | lower 95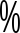 CI | upper 95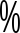 CI |
| 14 | 224 | 2 | 0.929 | 0.01658 | 0.897 | 0.962 |
| 15 | 222 | 2 | 0.921 | 0.01746 | 0.887 | 0.956 |
| 16 | 220 | 1 | 0.917 | 0.01787 | 0.882 | 0.952 |
| 19 | 219 | 1 | 0.912 | 0.01827 | 0.877 | 0.949 |
| 20 | 218 | 1 | 0.908 | 0.01866 | 0.872 | 0.945 |
| 27 | 217 | 1 | 0.904 | 0.01904 | 0.867 | 0.942 |
