# Supplementary material for: Construction and validation of a robust prognostic model based on immune features in sepsis
Source: Front Immunol. 2022 Dec 2;13:994295. doi: 10.3389/fimmu.2022.994295 (PMC9756843; doi:10.3389/fimmu.2022.994295)
Supplement: Supplementary file 10 [file Table_9.docx]

Table S9. Univariate independent prognostic analysis of sepsis clinical characteristics based on risk model.

| id | HR | HR.95L | HR.95H | pvalue |
| --- | --- | --- | --- | --- |
| Age | 1.023691043 | 1.003794605 | 1.043981852 | **0.019378338** |
| Gender | 0.969532853 | 0.588691629 | 1.596751009 | 0.90325486 |
| Diabetes | 1.146094282 | 0.640883328 | 2.049565102 | 0.64566774 |
| ICUA | 1.47353392 | 0.799362631 | 2.71629187 | 0.214119395 |
| Endotype class | 0.867266594 | 0.665173147 | 1.130760238 | 0.292762124 |
| RiskScore | 1.225316733 | 1.158553976 | 1.295926757 | **1.17E-12** |
