# Supplementary material for: Construction and validation of a robust prognostic model based on immune features in sepsis
Source: Front Immunol. 2022 Dec 2;13:994295. doi: 10.3389/fimmu.2022.994295 (PMC9756843; doi:10.3389/fimmu.2022.994295)
Supplement: Supplementary file 11 [file Table_10.docx]

Table S10. Multivariate independent prognostic analysis of sepsis clinical characteristics based on risk model.

| id | HR | HR.95L | HR.95H | pvalue |
| --- | --- | --- | --- | --- |
| Age | 1.032005927 | 1.009622328 | 1.054885776 | **0.004863926** |
| Gender | 0.937935113 | 0.564648822 | 1.557998957 | 0.804546872 |
| Diabetes | 0.955888636 | 0.523148162 | 1.746585672 | 0.88337641 |
| ICUA | 1.138956826 | 0.597758895 | 2.170143616 | 0.692422196 |
| Endotype class | 1.003057797 | 0.767796191 | 1.310406271 | 0.982138241 |
| RiskScore | 1.242291691 | 1.1711294 | 1.317778075 | **5.65E-13** |
