# Supplementary material for: Construction and validation of a robust prognostic model based on immune features in sepsis
Source: Front Immunol. 2022 Dec 2;13:994295. doi: 10.3389/fimmu.2022.994295 (PMC9756843; doi:10.3389/fimmu.2022.994295)
Supplement: Supplementary file 12 [file Table_11.docx]

Table S11. Relationships between the IRGs in risk models and the clinical characteristics in sepsis.

| id | Age | | Gender | | Diabetes | | ICUA | | Endotype class | |
| --- | --- | --- | --- | --- | --- | --- | --- | --- | --- | --- |
|  | t | pvalue | t | pvalue | t | pvalue | t | pvalue | t | pvalue |
| ADRB2 | -0.56 | 0.576 | 1.245 | 0.214 | 0.046 | 0.963 | 0.983 | 0.33 | -2.086 | **0.039** |
| CD1D | -0.725 | 0.469 | 1.949 | 0.052 | -0.366 | 0.715 | 2.331 | **0.023** | -4.269 | **3.66E-05** |
| CD74 | 1.052 | 0.294 | 0.422 | 0.673 | -0.171 | 0.864 | 0.878 | 0.384 | -4.413 | **1.83E-05** |
| CETP | -0.6 | 0.549 | -1.386 | 0.167 | 0.79 | 0.432 | -0.124 | 0.902 | -0.888 | 0.376 |
| ELANE | -0.918 | 0.36 | 1.27 | 0.205 | -1 | 0.32 | -0.803 | 0.426 | 2.368 | **0.019** |
| FYN | 0.068 | 0.946 | 0.029 | 0.977 | -1.157 | 0.25 | 1.118 | 0.268 | -2.405 | **0.017** |
| GNLY | 1.356 | 0.176 | -0.81 | 0.419 | -1.026 | 0.307 | -0.324 | 0.747 | -2.25 | **0.026** |
| HLA-DRA | 0.393 | 0.694 | 0.648 | 0.518 | -0.503 | 0.616 | 0.494 | 0.623 | -1.89 | 0.06 |
| IL16 | -0.218 | 0.828 | -0.015 | 0.988 | -0.293 | 0.77 | 0.736 | 0.465 | -5.599 | **8.67E-08** |
| IL17RA | -0.799 | 0.425 | -0.001 | 0.999 | 0.421 | 0.675 | 0.435 | 0.666 | -10.643 | **9.04E-19** |
| IL1R2 | -1.526 | 0.128 | -0.722 | 0.471 | 0.367 | 0.714 | -1.422 | 0.16 | -1.593 | 0.114 |
| LTB | 0.789 | 0.431 | -1.176 | 0.241 | -0.701 | 0.485 | 0.318 | 0.752 | -1.928 | 0.056 |
| MPO | -0.429 | 0.668 | 1.704 | 0.09 | -0.926 | 0.357 | -0.18 | 0.858 | 2.274 | **0.024** |
| PLXNC1 | -1.091 | 0.276 | 0.608 | 0.544 | -1.221 | 0.224 | 0.907 | 0.368 | -6.25 | **9.76E-09** |
| PSME1 | 0.671 | 0.503 | -0.701 | 0.484 | 0.448 | 0.655 | 1.356 | 0.181 | -5.736 | **4.13E-08** |
| TAP2 | 1.343 | 0.18 | 0.194 | 0.846 | 0.481 | 0.631 | 0.841 | 0.404 | -7.306 | **5.52E-12** |
| TFRC | 0.424 | 0.672 | 0.711 | 0.478 | 0.219 | 0.827 | -1.265 | 0.211 | 8.424 | **5.93E-14** |
| THBS1 | -0.337 | 0.737 | -0.157 | 0.875 | -0.167 | 0.868 | -1.715 | 0.093 | 1.38 | 0.17 |
| TNFRSF10B | -0.155 | 0.877 | 0.601 | 0.549 | -0.982 | 0.328 | 1.146 | 0.257 | -4.65 | **8.04E-06** |
| TNFSF12 | -0.161 | 0.873 | -0.408 | 0.683 | -0.556 | 0.58 | 1.093 | 0.279 | -4.588 | **9.01E-06** |
| TRBV9 | -0.034 | 0.973 | -0.782 | 0.435 | -0.588 | 0.558 | 0.92 | 0.361 | 0.347 | 0.729 |
| DEFA4 | -1.023 | 0.307 | 0.62 | 0.536 | -1.181 | 0.241 | -0.636 | 0.528 | 3.846 | **1.70E-04** |
| riskScore | 1.023 | 0.307 | -0.878 | 0.381 | 0.726 | 0.469 | -1.251 | 0.216 | 3.122 | **0.002** |
